# Artificial intelligence models for predicting acute kidney injury in the intensive care unit: a systematic review of modeling methods, data utilization, and clinical applicability

**DOI:** 10.1093/jamiaopen/ooaf065

**Published:** 2025-07-03

**Authors:** Tongyue Shi, Yu Lin, Huiying Zhao, Guilan Kong

**Affiliations:** National Institute of Health Data Science, Peking University, Beijing 100191, China; Institute for Artificial Intelligence, Peking University, Beijing 100871, China; Advanced Institute of Information Technology, Peking University, Hangzhou, Zhejiang 311215, China; Department of Twin Research and Genetic Epidemiology, King’s College London, London SE1 7EH, United Kingdom; Department of Critical Care Medicine, Peking University People’s Hospital, Beijing 100044, China; National Institute of Health Data Science, Peking University, Beijing 100191, China; Institute for Artificial Intelligence, Peking University, Beijing 100871, China; Advanced Institute of Information Technology, Peking University, Hangzhou, Zhejiang 311215, China

**Keywords:** acute kidney injury, intensive care unit, prediction modeling, artificial intelligence, machine learning, systematic review

## Abstract

**Objectives:**

Acute kidney injury (AKI) is common in intensive care unit (ICU) patients and is associated with high mortality, prolonged ICU stays, and increased costs. Early prediction is crucial for timely intervention and improved outcomes. Various prediction models, including machine learning, deep learning, and dynamic prediction frameworks, have been developed, but their modeling approaches, data utilization, and clinical applicability require further investigation. This review comprehensively assesses the modeling methods, data utilization strategies, and clinical applicability of AKI prediction models in the ICU, identifies current challenges, and proposes future research directions.

**Materials and Methods:**

A systematic search was conducted in PubMed, Embase, Scopus, Web of Science, IEEE Xplore, and ACM Digital Library up to December 12, 2024. Studies were included if they reported AKI prediction models using ICU-specific data, included at least 2 predictors, and evaluated model performance. Extracted data included study characteristics, model details, data sources, performance metrics, and validation methods. The risk of bias was assessed using PROBAST (Prediction Model Risk of Bias Assessment Tool), and the reporting quality was evaluated using the TRIPOD (Transparent Reporting of a multivariable prediction model for Individual Prognosis Or Diagnosis) guideline.

**Results:**

From 1305 screened studies, 47 met the inclusion criteria. Models ranged from machine learning to advanced deep learning techniques. Only 14 studies conducted external validation. Most studies (*n* = 44) had a high risk of bias, particularly in generalizability and clinical applicability.

**Discussion:**

Although AI models have shown promise in predicting AKI in ICU settings, key challenges remain. These include limited external validation, lack of dynamic modeling, insufficient interpretability, and poor consideration of clinical integration. Different study designs, prediction windows, and data sources also hinder model comparability.

**Conclusions:**

Future research should prioritize dynamic, interpretable, and externally validated models. These efforts are critical to bridge the gap between model development and clinical implementation and to enhance the real-world applicability of AI in AKI prediction.

## Introduction

Acute kidney injury (AKI) is a clinical syndrome with a rapid decline in renal function, manifesting as decreased glomerular filtration rate (GFR), elevated levels of creatinine and urea nitrogen, and disturbances in fluid, electrolyte, and acid–base balance, which in severe cases can lead to multisystem complications.^1^^,^^2^ AKI is prevalent in hospitalized patients, with an incidence of 10%-15%, rising to 50% in the intensive care units (ICUs).^1^^,^^2^ Approximately 10% of AKI patients require renal replacement therapy (RRT), with a hospital mortality rate reaching 23% and exceeding 50% for severe cases.^3–6^ Elevated risks of chronic kidney disease and end-stage renal disease were observed in post-AKI patients, increasing the long-term burden on individuals and health-care systems.^1^^,^^2^^,^^6^ Additionally, prolonged ICU stays in AKI patients increase hospital costs, particularly in cases requiring RRT, accounting for 15%-25% of total hospital expenditures.^2^^,^^6^^,^^7^

The high incidence, mortality, and economic burden of AKI in the ICU underscore the urgent need for early prediction techniques. Current diagnostic criteria, such as the Kidney Disease: Improving Global Outcomes (KDIGO) guidelines,^1^^,^^2^^,^^8^ rely on serum creatinine and urine output changes, often detectable only after kidney injury, missing the optimal time window for early intervention. Early prediction of AKI risk offers opportunities to mitigate this delay, enabling timely interventions.^9–11^ Moreover, early prediction facilitates personalized treatment, optimizes healthcare service utilization, and reduces healthcare costs.^4^^,^^11^

Advances in artificial intelligence (AI), particularly machine learning (ML) and deep learning (DL), have revolutionized early prediction in critical care.^12^ Unlike traditional statistical methods, which often struggle with nonlinear relationships and high-dimensional data, AI models excel in handling complex ICU electronic health records (EHRs) data, capturing temporal patterns, and processing heterogeneous inputs.^10^^,^^13–17^ These strengths make AI a promising tool for dynamic risk assessment and early warning systems in AKI management.^12^^,^^18^^,^^19^ AI models provide actionable insights to clinicians, aiming to reduce AKI incidence and complications, enhance patient outcomes, and alleviate ICU resource burdens.^6^^,^^20^ However, there are still barriers to the broader adoption of these models, including challenges in model generalizability and integration into routine clinical workflows.^21^

This systematic review comprehensively examines the development and application of AI-based AKI prediction models in ICU settings, focusing on modeling methods, data utilization, and clinical applicability. Unlike prior reviews,^4^^,^^11^^,^^14^^,^^22^ which lacked methodology evaluation, this review highlights strengths, limitations, and practical challenges in deploying prediction models. By identifying gaps in prediction modeling and clinical implementation, we aim to provide practical recommendations for advancing the robustness and applicability of AKI prediction models in real-world settings.

## Methods

### Literature search

This systematic review followed the Preferred Reporting Items for Systematic Reviews and Meta-Analyses (PRISMA) guidelines.^23^^,^^24^ The protocol was preregistered in the International Database of Prospectively Registered Systematic Reviews (PROSPERO) (registration ID: CRD42024625888).^25^ Eligible studies were identified by searching 6 databases, including PubMed, Embase, Scopus, Web of Science, IEEE Xplore, and ACM Digital Library, from their inception to December 12, 2024. The search strategy was designed similarly to previous studies,^4^^,^^11^^,^^14^^,^^22^ and targeted terms related to ICU, AKI, early prediction, and AI models. A combination of medical subject headings and free-text terms ensured comprehensive retrieval of relevant literature. The detailed search terms and strategies are provided in Table S1.

### Inclusion and exclusion criteria

We included studies using the following criteria: (1) focused on adult patients admitted to ICUs; (2) used routinely collected data from EHRs in real-world local hospitals or publicly available ICU databases; and (3) developed prediction models for the occurrence of AKI using AI methods. The exclusion criteria were as follows: (1) validation studies that did not include prediction model development; (2) nonresearch documents, such as conference abstracts, patents, books, and editorials; (3) unpublished literature without formal peer review, such as preprint articles; and (4) articles not written in English.

### Study selection and data extraction

Following the search strategies, relevant studies were identified through titles and abstracts. Two authors independently screened the literature using the predefined inclusion and exclusion criteria. The screening process consisted of 2 stages: first, titles were reviewed to exclude irrelevant studies, and subsequently, abstracts and full texts were evaluated to determine the final set of eligible studies. Discrepancies between reviewers were resolved through group discussions to achieve consensus.

A standardized template was used during data extraction to ensure consistency and comprehensiveness. Extracted information included study design, data sources, patient populations, prediction models, and performance metrics. The extracted data were systematically categorized into predefined groups, such as study characteristics, prediction methodologies, and performance outcomes, to facilitate analysis.

### Quality assessment and critical appraisal

We assessed the included studies based on their study characteristics. The performance of the models was evaluated primarily using the area under the receiver operating characteristic curve (AUROC), which reflects overall discriminative ability.^26^ For studies employing multiple predictive algorithms, we reported the AUROC of the best-performing model. In addition to AUROC, we also extracted other performance metrics where available, such as sensitivity, specificity, positive predictive value (PPV), negative predictive value (NPV), and F1 score.

We evaluated the risk of bias and applicability of the included studies using the Prediction Model Risk of Bias Assessment Tool (PROBAST), which is specifically designed for evaluating prediction model studies.^27^^,^^28^ PROBAST consists of 2 main components: Risk of Bias and Applicability. The Risk of Bias component includes 4 domains: Participants, Predictors, Outcome, and Analysis, while Applicability includes 3 domains: Participants, Predictors, and Outcome. Each domain was assessed using a 3-level grading system: “+” indicates low risk of bias or low concern for applicability, “−” indicates high risk or high concern, and “?” indicates unclear risk or concern. The detailed assessment criteria for each domain were as follows. For Risk of Bias: (1) Participants were rated “+” if the study included a clearly defined and appropriate ICU population; (2) Predictors were rated “+” if the selection of variables was clearly explained (eg, based on clinical knowledge, literature, or algorithms), and “?” if the selection method was unclear; (3) Outcome was rated “+” when AKI was appropriately defined and measured; (4) Analysis was rated “−” if the study lacked model validation or calibration, and “?” if there was no mention of handling missing data or if the study was limited to single-center data. For Applicability: (1) Participants were rated “−” if the study used single-center data; (2) Predictors were rated “?” if the study did not address clinical interpretability (eg, black-box models without interpretability, or clear variable definitions); (3) Outcome was generally rated “+” if AKI was defined consistently with clinical standards. For each included study, we assessed the corresponding reporting quality using the Transparent Reporting of a multivariable prediction model for Individual Prognosis Or Diagnosis (TRIPOD) guidelines.^29^

## Results

### Study characteristics

**Figure 1.**
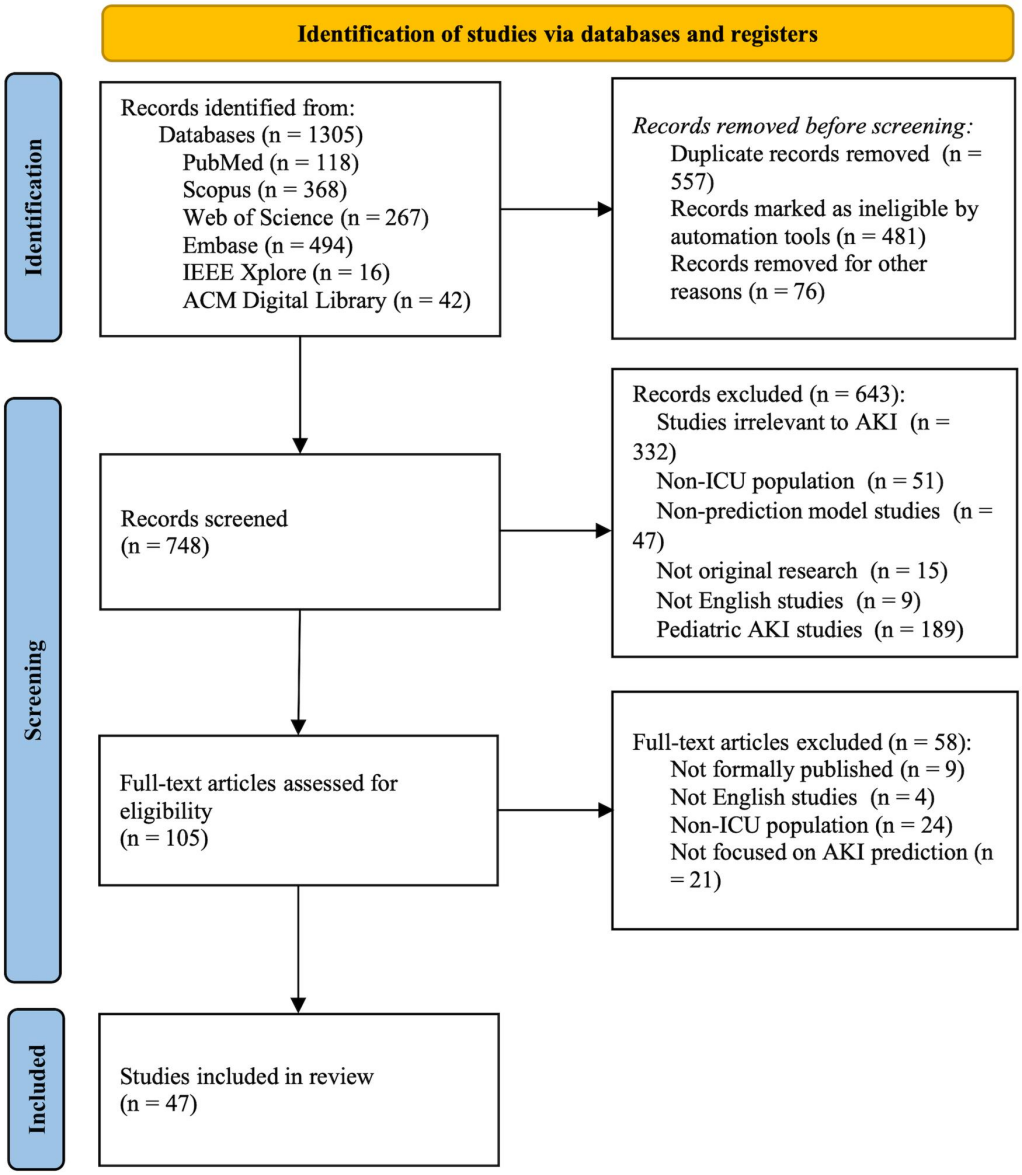
Flow chart of the study selection process.

A total of 1305 articles were identified from 6 databases, and 47 studies were included in this systematic review following the PRISMA guidelines.^5^^,^^9^^,^^30–74^ The literature searching process is illustrated in Figure 1. The key characteristics of the included studies, including study design, data sources, study populations, and AKI incidence, are summarized in Table S2.

Of all the studies included, 31 were single-center studies, and 16 were multicenter studies. Most studies employed retrospective designs (*n* = 45), while only 2 used prospective designs. Geographically, most studies originated from China (*n* = 31) and the United States (*n* = 7), with other contributions from Europe (*n* = 6), India (*n* = 2), and Japan (*n* = 1).

The study populations primarily consisted of general ICU patients (*n* = 29), while others targeted specific subgroups, including sepsis patients (*n* = 5), patients with cirrhosis (*n* = 2), acute pancreatitis (*n* = 2), acute cerebrovascular disease (*n* = 1), acute myocardial infarction (*n* = 1), rhabdomyolysis (*n* = 1), traumatic brain injury (*n* = 1) and so on. Sample sizes in the training sets varied widely, ranging from 394 to over 403 000 participants. These studies utilized diverse data sources, and publicly available databases were frequently used. The Medical Information Mart for Intensive Care (MIMIC)-III^75^ (*n* = 23), MIMIC-IV^76^ (*n* = 15), and eICU Collaborative Research Database (eICU-CRD)^77^ (*n* = 10) were the most common data sources, alongside other open-access datasets. Besides, 15 studies relied on real-world data from local hospitals.

The majority of studies defined AKI using the KDIGO^78^ (*n* = 42), and a smaller number adopted the Acute Kidney Injury Network (AKIN)^79^ (*n* = 3), International Classification of Diseases (ICD) code-based identification (*n* = 1), and Acute Disease Quality Initiative^80^ (*n* = 1). The remaining studies utilized tailored definitions based on local or specific research needs. The observed AKI incidence in the training datasets ranged from as low as 3.03% to as high as 85.03%, with most studies reporting incidence between 10% and 50%.

### Modeling methods

The prediction model characteristics of the included studies are displayed in Table 1. Machine learning models such as eXtreme Gradient Boosting (XGBoost), random forest (RF), Naive Bayes (NB), and support vector machines (SVM) were widely applied.^81^ XGBoost, the most frequently used ML algorithm, was identified as the best-performing model in 10 studies. Similarly, LightGBM and RF were the best models in 3 studies. With rapid advancement in DL techniques, several studies utilized DL methods for AKI prediction. Among these, 9 studies reported DL models, such as long short-term memory (LSTM) networks and convolutional neural networks (CNNs), as the best-performing models. Notably, 37 studies developed multiple models for comparison, aiming to identify an optimal approach. Logistic regression (LR) was utilized in 30 studies, RF in 25, XGBoost in 23, SVM in 18, and NB in 10. Among DL models, artificial neural network (ANN) was employed in 10 studies, LSTM in 7, and CNN in 4. Additionally, ensemble models were adopted in several studies and often demonstrated superior performance by integrating individual algorithms for improved predictive accuracy.

**Table 1.** Characteristics of models for AKI prediction in the selected studies.

Study	Modeling algorithm	Best model	Validation strategy	AUROC of the best model	Other metrics to measure performance	Dynamic prediction	Interpretability	Predicted time window	Model presentation form	Model calibration
Zhang et al.^30^	XGBoost, LR, SVM, RF and NB	XGBoost	Internal validation (7:3 random sampling and 5-fold cross-validation)	0.767 (0.721-0.795)	Sensitivity: 0.633, Specificity: 0.738, F1 score: 0.648, PPV: 0.909, NPV: 0.466	No	SHAP	First 24 hours after ICU admission	Not reported	Yes
Yang et al.^31^	DeepAKI Neural Network, LSTM, GBDT, and LR	DeepAKI Neural Network	Internal validation and external validation	0.799 (0.791-0.806) (internal); 0.763 (0.755-0.771) and 0.676 (0.668-0.684) (external)	Sensitivity: 0.750, Specificity: 0.701, PPV: 0.404, NPV: 0.971	Yes	SHAP	Predicting AKI risk in the next 24 hours	Not reported	Not reported
Tu et al.^32^	LR	LR	Internal validation (2:1 random sampling)	0.797	Not reported	No	Not reported	Risk of AKI within 7 days after ICU admission	Nomogram	Yes
Tan et al.^33^	LSTM, LR, XGBoost, ClinicalBERT, BioMedBERT and multimodal model	Multimodal model	Internal validation (4:1 random sampling)	0.888	Accuracy: 0.860, Precision: 0.642, Recall: 0.693, AUPRC: 0.727	No	SHAP	AKI diagnosis or CRRT requirement prediction within 12 hours after an initial 6-hour data collection window	Not reported	Not reported
Sun et al.^34^	RF, SVM, NB, XGBoost, LR, and ANN	XGBoost	Internal validation (10-fold cross-validation) and external validation	0.902 (internal); 0.889 (external)	Cutoff: 0.336, Accuracy: 0.815, Sensitivity: 0.802, Specificity: 0.820, PPV: 0.685, NPV: 0.916, F1 score: 0.687	No	SHAP	Early prediction of AKI within 7 days for critically ill patients	Website tool	Not reported
Shi et al.^35^	LightGBM, XGBoost, RF, DT, ANN, SVM, LR, and both LightGBM and XGBoost models	LightGBM	No validation	0.801	Accuracy: 0.829, F1 score: 0.902	No	Not reported	Two days after ICU admission	Not reported	Not reported
Lyu et al.^36^	GBDT (time-stacked, history-based, and snapshot-based variants), and LSTM	Time-stacked GBDT	Internal validation (4:1 random sampling) and external validation	Not reported	AUPRC: 0.657, Precision: 0.8, Recall: 0.492	Yes	SHAP	Each 48-hour interval after ICU admission	Not reported	Yes
Lu et al.^37^	LR, XGBoost, LightGBM, RF, AdaBoost, GNB, MLP, SVM, and KNN	XGBoost	Internal validation (7:3 random sampling) and external validation	0.940 (internal); 0.951 (validation)	Accuracy: 0.999, Sensitivity: 1.000, Specificity: 1.000, PPV: 0.999, NPV: 1.000, F1 score: 1.000	No	SHAP	Throughout ICU stay	Not reported	Yes
Liu et al.^38^	ANN and LR	ANN	Internal validation (7:3 random sampling)	0.938	Sensitivity: 0.940, Specificity: 0.810, PPV: 0.798, NPV: 0.940	No	Not reported	Throughout ICU stay	Not reported	Yes
Lin et al.^39^	GBM, GLM, KNN, NB, NNET, RF, and SVM	GBM	Internal validation (7:3 random sampling)	0.814 (0.763-0.865)	Not reported	No	Variable importance of features included in gradient boosting machine algorithm	Seven days after ICU admission	Not reported	Yes
Li et al.^40^	LR, XGBoost, LightGBM, MLP, RF, and KNN	LightGBM	Internal validation (10-fold cross-validation) and external validation	0.853 (0.841- 0.865) (internal); 0.755 (0.699-0.811) (external)	Sensitivity: 0.788, Specificity: 0.761, PPV: 0.522, NPV: 0.915	No	SHAP	Throughout ICU stay	Online apps with machine learning algorithms	Yes
Zheng et al.^41^	LR, RF, GBM, KNN, ANN, SVM, and NB	The best 4 performing models (RF, GBM, KNN, and ANN) and then constructed a new model (DC-AKI) using weighted voting	Internal validation (7:3 random sampling) and external validation	0.805 (0.763-0.845) (internal); 0.772 (0.745-0.801) (external)	Not reported	No	Not reported	First 24 hours of ICU admission	Risk score model	Yes
Zhang et al.^42^	ALSTM and LightGBM	ALSTM	Internal validation (7:3 random sampling)	0.901, 0.890 and 0.877 in the 6-, 12- and 24-hour model	Accuracy: 0.818, 0.806 and 0.797 in the 6-, 12- and 24-hour model	Yes	Visualization of attention weights for lab measurements	Prediction windows of 2, 6, 12, and 24 hours	Not reported	Not reported
Wu et al.^43^	C5.0, SVM, Bayes, XGBoost, and the ensemble model	The ensemble model	Internal validation (7:3 random sampling)	0.845 (0.78-0.91)	Not reported	No	iBreakdown	24 hours after ICU admission	Not reported	Yes
Wu et al.^44^	SVM, RF, CNN, LSTM and LSTM with self-attention layer (LSTMATT)	LSTMATT	Internal validation (training, validation and test data according to a proportion of 8:1:1)	0.89	Not reported	Yes	SHAP	Consecutive 12-hour windows predicting AKI within the next *n* hours	Not reported	Not reported
Shi et al., 2023^9^	LR, XGBoost, and XGBoost with severity scores (SOFA, OASIS, and APS III)	XGBoost with severity scores	Internal validation (7:3 random sampling) and external validation	0.87 (internal); 0.84 (external)	Accuracy: 0.870, Precision: 0.860, Recall: 0.720, AUC: 0.890, AUPRC: 0.850, F1 score: 0.860	No	SHAP	24 hours after ICU admission	Not reported	Yes
Persson et al.^45^	NAVOY-AKI	NAVOY-AKI	Internal validation	0.91	AUPRC: 0.750, Accuracy: 0.840, Sensitivity: 0.800, Specificity: 0.850, PPV: 0.610	Yes	SHAP	First 4 hours of data, predicting AKI 12 hours before onset	Not reported	Yes
Peng et al.^46^	ANN, NB, LR, GBM, AdaBoost, RF, BT, and XGBoost	RF	Internal validation (5-fold cross-validation) and external validation	Not reported (internal); 0.819 (0.783-0.851) (external)	Not reported	No	SHAP	Less than 24 hours after ICU admission	Not reported	Yes
Pan et al. 2023^5^	SVM, LR, RF, and XGBoost	XGBoost	Internal validation (4:1 random sampling)	0.892	Accuracy: 0.809, Specificity: 0.856, Precision: 0.842, Recall: 0.759, F1 score: 0.799	Yes	SHAP	3, 6, 9, and 12 hours	Not reported	Not reported
Luo et al.^47^	XGBoost, LR, SVM, RF, ANN, Gradient Boosting, and LightGBM	XGBoost	Internal validation (7:3 random sampling)	0.849	Not reported	No	Not reported	24 hours after ICU admission	Not reported	Not reported
Jiang et al.^48^	LR	LR	Internal validation (7:3 random sampling)	0.82 (0.79-0.86)	Not reported	No	Not reported	24 hours after ICU admission	Nomogram	Yes
Huang et al.^49^	LR and RF	RF	Internal validation and External validation	AKI-23: 0.82 (0.76-0.88) (internal), 0.80 (0.76, 0.84) (external); AKI-3: 0.89 (0.84-0.95) (internal); 0.80 (0.73-0.86)	AKI-23: Sensitivity: 0.57 (0.55-1.00), Specificity: 0.90 (0.42-0.94), PPV: 0.39 (0.17-0.53), NPV: 0.95 (0.94-1.00), Classification threshold: 23.5; AKI-3: Sensitivity: 0.79 (0.67-1.00), Specificity: 0.86 (0.62-0.92), PPV: 0.27 (0.14-0.40), NPV: 0.98 (0.98-1.00), Classification threshold: 11.9	No	Not reported	AKI-23: Prediction of the occurrence of stage 2 or 3 AKI during the first week of ICU stay after the initial 12 hours of MV; AKI-3: Prediction of the first occurrence of stage 3 AKI during the first week of ICU stay after the initial 12 hours of MV	Not reported	Yes
Begum et al.^50^	Stacked LSTM model and GBT	Stacked LSTM model	Internal validation	Window size: 12 hours: 0.924, Window size: 24 hours: 0.9263	For a 12-hour window size: Accuracy: 0.924, Sensitivity: 0.862, Specificity: 0.902, LR+: 4.28, LR−: 0.24; For a 24-hour window size: Accuracy: 0.926, Sensitivity: 0.871, Specificity: 0.902, LR+: 5.06, LR−: 0.20	Yes	Not reported	First 12 and 24 hours of ICU admission	Not reported	Not reported
Yue et al.^51^	LR, KNN, SVM, Decision Tree, RF, XGBoost, and ANN	XGBoost	Internal validation (10-fold cross-validation)	0.817	Not reported	No	Feature importance in XGBoost models	During the first 24 hours of ICU stay	Not reported	Yes
Zhang et al.^52^	SVM, RF, NNET, XGboost, and ensemble model	Ensemble model	Internal validation (5-fold cross-validation) and external validation	0.774-0.788 (internal); 0.756-0.813 (external)	Not reported	No	LIME, SHAP, and iBreakDown	48, 36, 24, and 12 hours before AKI onset	An online risk calculator on the website	Not reported
Zeng et al.^53^	LR, XGBoost, RNN and Sequence Transformer	Sequence Transformer	Internal validation and external validation	2-day AKI: 0.787 (0.786, 0.789) (internal); 0.656 (0.652, 0.659) (external); 7-day AKI: 0.795 (95%CI: 0.792, 0.797) (internal); 0.672 (0.668, 0.676) (external)	2-day AKI: Accuracy: 0.702, F1-score: 0.571, AUPRC: 0.560; 7-day AKI: Accuracy: 0.712, F1-score: 0.630, AUPRC: 0.650	Yes	Not reported	Two and 7 days	Not reported	Not reported
Zhang et al.^54^	XGBoost, AdaBoost, RF, LR, and MLP	XGBoost	Internal validation (2:1:1 random sampling) and external validation	0.880 (95%CI: 0.831-0.929) (internal); 0.780 (0.731–0.829) (external)	Not reported	No	XGBoost, AdaBoost, and random forest classification models to acquire the relative importance score for each feature individually and then took the average from all 3 models	During the first 24 hours of ICU stay	Not reported	Yes
Wen et al.^55^	LR	LR	Internal validation (4:1 random sampling)	0.8289	Not reported	No	Not reported	7 days	Nomogram	Yes
Vagliano et al.^56^	LSTM+Clinical variables, LSTM+Clinical notes, LSTM+Variables + notes, LSTM+Variables + notes + UMLS syn., LSTM+Variables + notes + SNOMED syn., and LSTM+Variables + notes + SNOMED parent-child	LSTM+Clinical variables	Internal validation (8:1:1 random sampling)	0.899	Not reported	No	Not reported	48 hours	Not reported	Yes
Gao et al.^57^	LR, RF, LightGBM, XGBoost, and Ensemble Model (combining RF, LightGBM, and XGBoost)	Ensemble Model	Internal validation (4:1 random sampling)	24-hour prediction: 0.923; 48-hour prediction: 0.902; 72-hour prediction: 0.895	For 24-hour prediction: F1 score: 0.915, Precision: 0.910, Recall: 0.924; For 48-hour prediction: F1 score: 0.893, Precision: 0.911, Recall: 0.892; For 72-hour prediction: F1 score: 0.878, Precision: 0.893, Recall: 0.861	Yes	SHAP	24, 48, and 72 hours	Not reported	Not reported
Begum et al.^58^	GBT and SOFA scoring system	GBT	Internal validation (10-fold cross-validation)	At AKI onset: 0.890 (0.867-0.878); 12 hours before onset: 0.821 (0.792-0.809); 24 hours before onset: 0.850 (0.785-0.804)	Onset: Sensitivity: 0.81, Specificity: 0.75, Accuracy: 0.863; 12 hours before onset: Sensitivity: 0.77, Specificity: 0.62, Accuracy: 0.76; 24 hours before onset: Sensitivity: 0.83, Specificity: 0.56, Accuracy: 0.82	Yes	SHAP	AKI at onset, 12 hours before, and 24 hours before onset	Not reported	Not reported
Cai et al.^59^	RF, XGBoost, NB, SVM, DT, and LR	RF	Internal validation (7:3 random sampling) and external validation	0.733 (0.695-0.770) (internal); 0.781 (95% CI: 0.750-0.811) (external)	Accuracy: 0.735, Sensitivity: 0.748, Specificity: 0.698	No	SHAP	During ICU stay	Not reported	Yes
Qian et al.^60^	LR, RF, SVM, LightGBM, XGBoost, and CNN	LightGBM	Internal validation (4:1 random sampling)	0.905	F1 score: 0.897, Recall: 0.836, Precision: 0.971	No	Feature importance ranked via Gini index and permutation methods	72 hours after ICU admission	Not reported	Not reported
Luo et al.^61^	LR, RF, SVM, ANN, and XGBoost	LR and ANN	Internal validation (7:3 random sampling)	0.76 (0.74-0.78)	Sensitivity: 0.630, Specificity: 0.760, PPV: 0.830, NPV: 0.530	No	Feature importance derived via XGB and LASSO	AKI persistence classification beyond 48 hours postdiagnosis	Not reported	Yes
Le et al.^62^	CNN, XGBoost, and SOFA scoring system	CNN	Internal validation (9:1 random sampling)	0.86	Sensitivity: 0.804, Specificity: 0.763, PPV: 0.236, NPV: 0.975, F1 score: 0.360	Yes	Incorporates clinical notes using Doc2Vec for unstructured data embedding; feature importance analysis via permutation methods	Up to 48 hours before AKI onset	Not reported	Not reported
Gong et al.^63^	LR, RF, XGBoost, and a voting ensemble method combining LR and RF	XGBoost	Internal validation (4:1 random sampling)	0.774 (0.748-0.789)	F1 score: 0.500, Precision: 0.326, Recall: 0.675, Accuracy: 0.759	Yes	SHAP	AKI prediction within 72 hours after ICU admission	Not reported	Yes
Chiorean et al.^64^	GBDT, and SVM	GBDT	Internal validation (5-fold cross-validation)	0.676	Precision: 0.450, Recall: 0.500, F1 score: 0.480	Yes	GBDT with feature importance ranking using mean decrease in impurity (MDI)	7 days following ICU admission	Not reported	Not reported
Alfieri et al.^65^	LR and deep learning (CNN with highway connections)	Deep learning model (CNN with highway connections)	Internal validation with randomly selected training, validation, calibration, and test sets	0.89 ± 0.01	Sensitivity: 0.820, Specificity: 0.840, LR+: 5.0, LR−: 0.2	Yes	High interpretability through time-series visualization and probabilistic outputs	AKI prediction ≥12 hours before onset	Not reported	Yes
Wang et al.^66^	ETSM (XGBoost-based ensemble time-series model), NB, kNN, RF, and AdaBoost	ETSM	Internal validation (3:2 random sampling) and external validation	24-hour prediction: 0.81 (internal), 0.95 (external); 48-hour prediction: 0.78 (internal), 0.95 (external)	Sensitivity: 0.750 (24 hours), Sensitivity: 0.680 (48 hours)	No	Feature importance	24 and 48 hours before AKI onset	Not reported	Not reported
Rank et al.^67^	RNN	RNN	Internal validation with cross-validation and comparison to clinicians	0.893 (95% CI, 0.862-0.924)	Sensitivity: 0.851, Specificity: 0.840, Precision: 0.842, NPV: 0.850, Calibration: 0.370	Yes	Partial; dependent on feature importance in the RNN framework	7 days following surgery	Not reported	Yes
Matsuura et al.^68^	LR	LR	Internal validation	0.79 (0.77-0.81)	Sensitivity: 0.638, Specificity: 0.813, PPV: 0.330, NPV: 0.939	No	Interpretability due to simple scoring system based on clinical risk factors and serum creatinine changes	Persistent AKI occurrence within 1 week of ICU admission	A simple risk score	Yes
Zimmerman et al.^69^	LR, RF, and MLP	MLP	Internal validation (5-fold cross-validation)	0.796	Sensitivity: 0.694, Specificity: 0.753, PPV: 0.357, NPV: 0.926	No	Not reported	AKI prediction for the second- and third-day following ICU admission	Not reported	Not reported
Wang et al.^70^	XGBoost, NB, RF, kNN, SVM, and AdaBoost	XGBoost	Internal validation (10-fold cross-validation)	24-hour prediction: 0.80; 48-hour prediction: 0.77	Sensitivity: 0.680 (24 hours), Sensitivity: 0.650 (48 hours)	No	Interpretability due to decision tree structure in XGBoost-VM	24 hours ahead and 48 hours ahead	Not reported	Not reported
Parreco et al.^71^	GBT, LR, and deep learning	GBT	Internal validation (10-fold cross-validation)	0.834 ± 0.006	F1 score: 0.429, Accuracy: 0.939	No	GBT’s variable importance	Over 5 days	Not reported	Not reported
Sun et al.^72^	LR, RF, SVM, NB, and CNN	SVM	Internal validation (7:3 random sampling)	0.8352	Not reported	No	Examined feature importance in logistic regression via L2-regularization	72 hours post-ICU admission	Not reported	Yes
Chiofolo et al.^73^	RF	RF	Internal validation (7:3 random sampling)	0.949 (0.943-0.954)	Sensitivity: 0.920, Specificity: 0.680, PPV: 0.420, NPV: 0.970	Yes	Not reported	Entire ICU stay; AKI detected 6+ hours before clinical criteria in 30% of cases	Not reported	Yes
Mao et al.^74^	LR	LR	No validation	0.845	Sensitivity: 0.783, Specificity: 0.862	No	Not reported	During ICU stay	Not reported	Not reported

Abbreviations: AKI: acute kidney injury; ALSTM: attention-based long short-term memory; ANN: artificial neural network; BioMedBERT: biomedical Bidirectional Encoder Representations from Transformers; ClinicalBERT: clinical Bidirectional Encoder Representations from Transformers; CNN: convolutional neural network; DT: decision tree; ETSM: ensemble time-series model; GBDT: gradient-boosted decision trees; GBT: gradient-boosted trees; GNB, Gaussian Naive Bayes; KNN: k-nearest neighbors; LightGBM: light gradient boosting machine; LR: logistic regression; LSTM: long short-term memory; LSTMATT: LSTM with self-attention; MLP: multilayer perceptron; NB: Naïve Bayes; RF: random forest; RNN: recurrent neural network; SeT: Sequence Transformer; SVM: support vector machine; XGBoost: eXtreme Gradient Boosting.

The AUROC of prediction models ranged from 0.66 to 0.95 across the included studies. In addition to AUROC, sensitivity across studies ranged from 0.63 to 0.92, specificity ranged from 0.56 to 0.91, PPV ranged from 0.27 to 0.97, NPV ranged from 0.33 to 1.00, and F1 scores ranged from 0.36 to 0.92. Accuracy was also frequently reported and exceeded 0.75 in well-performing models.

Among the 47 included studies, 17 investigated dynamic prediction models. These models employed time-series components such as LSTM networks and gradient-boosted decision trees (GBDT).^36^^,^^50^^,^^56^ Prediction windows varied from short intervals (eg, 6-12 h) to longer durations (eg, 7 days).^42^ One LSTM-based model achieved an AUROC of 0.901 across multiple windows (2, 6, 12, and 24 h), while a time-stacked GBDT model also reported an AUROC of 0.901.^36^

Most studies (*n* = 44) utilized internal validation methods, employing techniques such as random sampling, cross-validation, or bootstrapping. The most common internal validation used a 7:3 train-test split. However, only 14 studies conducted external validation, and 13 studies conducted both internal and external validation.

Interpretability emerged as a critical component for bridging the gap between advanced predictive models and clinical practice. Over half of the studies (*n* = 33) employed interpretability methods, with Shapley Additive Explanations (SHAP)^82^ being the most widely used technique. Other studies utilized alternative methods, such as iBreakdown^83^ and Local Interpretable Model-Agnostic Explanations (LIME),^84^ to enhance interpretability. Despite these efforts, 14 studies either did not report interpretability methods or did not consider model interpretability.

### Data utilization

In Table S3, we summarized the feature selection methods, the number of predictors used, and the predictive features reported in the included studies. A variety of approaches were used for feature selection, including statistical methods like Least Absolute Shrinkage and Selection Operator (LASSO) and regression and recursive feature elimination (RFE).^85^ These methods aimed to identify the most relevant predictors of AKI onset in ICU settings. Among the included studies, most studies (*n* = 29) explicitly described their feature selection methods, while others relied on implicit selection approaches. Feature selection techniques ranged from traditional methods (eg, univariate analysis) to advanced ML-based strategies like RFE. The number of predictors varied widely, ranging from 8 to over 150 variables. Frequently identified predictors included demographic and clinical features, such as age, comorbidities, vital signs, and laboratory tests. The top predictive features reported across studies were serum creatinine, blood urea nitrogen, systolic blood pressure, heart rate, and age. Time-series features, such as trends in creatinine levels or urine output over time, were included in approximately one-third of the studies. Features derived from time-series analysis included changes in vital signs, laboratory tests, and urine output within specified time windows.

Missing data handling strategies varied across the included studies. Simple imputation techniques were most frequently used. Specifically, median or mean imputation was employed in 13 studies, while forward or last observation carried forward imputation appeared in 6 studies. A total of 14 studies excluded variables with more than a specified data missing rate, most commonly using thresholds of 20%-40%. Only 15 studies applied more advanced methods such as multiple imputation, Gaussian regression-based imputation, RF imputation, or generative adversarial networks. In contrast, 5 studies excluded patients with any missing data, and 6 studies did not report their missing data handling strategy.

### Risk of bias and clinical applicability

We assessed the risk of bias and applicability of the 47 included studies using the PROBAST. The domain-level results are summarized, with full details provided in Table 2. In the risk of bias assessment, all studies were rated as low risk in the Participants domain and the Outcome domain. In the Predictors domain, 29 studies were rated as low risk and 18 as unclear. In the Analysis domain, 9 studies were rated as low risk, 34 as high risk, and 4 as unclear. For applicability, 16 studies were rated as having low concern in the Participants domain and 31 as unclear. In the Predictors domain, 34 studies were rated as low concern and 13 as unclear. All studies were rated as having low concern in the Outcome domain. Overall, 4 studies were rated as having low risk of bias, 34 as high risk, and 9 as unclear. Regarding overall concern for applicability, 13 studies were rated as low, 31 as high, and 3 as unclear. A total of 3 studies were rated as having both low risk of bias and low concern for applicability across all domains. The detailed reporting quality of each included study assessed using the TRIPOD guideline is presented in Table S4.

**Table 2.** Risk of bias and applicability of acute kidney injury prediction models assessed using the Prediction Model Risk of Bias Assessment Tool.

Study	Risk of bias				Applicability			Overall	
	1. Participants	2. Predictors	3. Outcome	4. Analysis	1. Participants	2. Predictors	3. Outcome	Risk of bias	Applicability
Zhang et al.^30^	+	?	+	+	+	+	+	?	+
Yang et al.^31^	+	?	+	?	+	+	+	?	+
Tu et al.^32^	+	+	+	−	−	?	+	−	−
Tan et al.^33^	+	?	+	−	−	+	+	−	−
Sun et al.^34^	+	+	+	?	+	+	+	?	+
Shi et al.^35^	+	?	+	−	−	?	+	−	−
Lyu et al.^36^	+	+	+	+	+	+	+	+	+
Lyu et al.^37^	+	+	+	−	−	+	+	−	−
Lu et al.^38^	+	+	+	−	−	?	+	−	−
Lin et al.^39^	+	+	+	−	−	+	+	−	−
Li et al.^40^	+	?	+	+	+	+	+	?	+
Zheng et al.^41^	+	+	+	−	−	?	+	−	?
Zhang et al.^42^	+	?	+	−	−	+	+	−	−
Wu et al.^43^	+	+	+	−	−	+	+	−	−
Wu et al.^44^	+	+	+	−	−	+	+	−	−
Shi et al. ^9^	+	+	+	−	+	+	+	−	+
Persson et al.^45^	+	+	+	−	−	+	+	−	−
Peng et al.^46^	+	+	+	+	+	+	+	+	+
Pan et al. ^5^	+	+	+	−	−	+	+	−	−
Luo et al.^47^	+	+	+	−	−	?	+	−	−
Jiang et al.^48^	+	+	+	−	−	?	+	−	−
Huang et al.^49^	+	+	+	+	+	?	+	+	?
Begum et al.^50^	+	?	+	−	−	+	+	−	−
Yue et al.^51^	+	?	+	−	−	+	+	−	−
Zhang et al.^52^	+	+	+	?	+	+	+	?	+
Zeng et al.^53^	+	?	+	+	+	?	+	?	?
Zhang et al.^54^	+	?	+	+	+	+	+	?	+
Wen et al.^55^	+	+	+	−	−	?	+	−	−
Vagliano et al.^56^	+	?	+	−	−	?	+	−	−
Gao et al.^57^	+	+	+	−	−	+	+	−	−
Begum et al.^58^	+	+	+	−	−	+	+	−	−
Cai et al.^59^	+	+	+	+	+	+	+	+	+
Qian et al.^60^	+	?	+	−	−	+	+	−	−
Luo et al.^61^	+	+	+	−	−	+	+	−	−
Le et al.^62^	+	?	+	−	−	+	+	−	−
Gong et al.^63^	+	+	+	−	−	+	+	−	−
Chiorean et al. 2021^64^	+	?	+	−	−	+	+	−	−
Alfieri et al.^65^	+	?	+	+	+	+	+	?	+
Wang et al.^66^	+	+	+	?	+	+	+	?	+
Rank et al.^67^	+	+	+	−	−	+	+	−	−
Matsuura et al.^68^	+	+	+	−	+	+	+	−	+
Zimmerman et al.^69^	+	+	+	−	−	?	+	−	−
Wang et al.^70^	+	?	+	−	−	+	+	−	−
Parreco et al.^71^	+	+	+	−	−	+	+	−	−
Sun et al.^72^	+	+	+	−	−	+	+	−	−
Chiofolo et al.^73^	+	+	+	−	−	?	+	−	−
Mao et al.^74^	+	?	+	−	−	?	+	−	−

The plus symbol (+) indicates a low risk of bias (ROB) or low concern for applicability; the minus symbol (−) means high ROB or high concern for applicability; the question mark (?) implies unclear ROB or unclear concern for applicability.

### Summative analysis

Across the 47 included studies, models including LR, RF, XGBoost, and SVM were the most commonly used. Model performance was primarily evaluated using AUROC, while other metrics and calibration were less reported. Only 14 studies conducted external validation, and 33 applied interpretation tools, mainly SHAP. Feature selection methods varied, with LASSO and expert-driven approaches being the most common. Public ICU datasets were widely used. Overall, although progress in modeling approaches is evident, limitations in generalizability, transparency, and clinical translation remain.

## Discussion

This systematic review synthesized evidence from 47 studies that developed or evaluated AI models for predicting AKI in the ICU. The studies were analyzed across 3 dimensions: modeling approaches, data utilization, and clinical applicability. The majority relied on retrospective single-center cohorts and publicly available datasets, most notably MIMIC and eICU-CRD, which are predominantly based on US populations. This raises concerns regarding generalizability across health-care systems and demographic groups. Sample sizes also varied widely, from several hundred to over 400 000, potentially contributing to heterogeneity in model performance. Taken together, these findings highlight the methodological diversity and ongoing limitations in model robustness, generalizability, and clinical readiness.

Compared to previous reviews,^4^^,^^11^^,^^86^ this review provides a more profound and broader methodological evaluation. Earlier reviews primarily focused on the overall performance of AI models but lacked in-depth exploration of models from crucial methodological aspects, such as model calibration, predictor selection, and dynamic prediction capabilities. Although dynamic prediction models demonstrate clinical potential, their development remains limited, often relying on single-center datasets with inadequate validation. Additionally, this review emphasizes the differences in data utilization, particularly in integrating time-series data and implementing dynamic prediction capabilities, offering detailed directions for future improvement and clinical translation. The following detailed discussions are structured into 3 subsections: modeling methods, data utilization, and risk of bias and clinical applicability.

### Modeling methods

#### Modeling approaches and performance

The reviewed studies adopted diverse modeling approaches for AKI prediction in the ICU. Logistic regression, though widely used as a benchmark model due to its simplicity and interpretability, often underperformed in capturing the nonlinear, high-dimensional, and temporal characteristics of ICU data. In contrast, other ML algorithms, such as RF and XGBoost, were frequently applied for their ability to balance performance and interpretability, while DL models, especially LSTM and CNN, demonstrated strength in modeling sequential and time-series inputs. Ensemble models, which combine multiple algorithms, demonstrate superior performance compared to individual models.^52^^,^^57^

Many studies built multiple models in parallel and selected the best-performing approach, typically based on AUROC. Reported AUROC values ranged from 0.657 to 0.951, with nearly half of the studies achieving scores above 0.85. However, AUROC alone does not fully reflect clinical utility. Sensitivity and specificity, reported in roughly one-third of the studies, indicate substantial variation in diagnostic tradeoffs. F1 scores, PPV, and NPV further illustrated differences in model behavior. Despite these efforts, model calibration was seldom reported. Only a few studies presented calibration curves or Brier scores. This gap hampers the clinical trustworthiness of predicted probabilities.

Future studies should report a comprehensive set of metrics and consider the intended use-case scenario when selecting and evaluating prediction models. With the development of technology, large language models and multimodal models will be more widely used in the ICU for future.^87^

#### Dynamic prediction

Dynamic prediction models have become increasingly common approaches, particularly within ICU settings where patient status can fluctuate rapidly. Unlike static models that rely on a single time-point snapshot, dynamic models continuously update risk estimates based on time-series inputs, such as evolving trends in urine output, serum creatinine, or vital signs. This allows for the timely detection of deterioration and supports early clinical interventions.

Among the reviewed studies, 17 explicitly adopted dynamic prediction frameworks, utilizing temporal modeling strategies such as sliding window segmentation, recurrent neural networks (eg, LSTM), or time-stacked decision trees (eg, GBDT).^36^^,^^42^^,^^50^ These models demonstrated strong predictive performance, with several reporting AUROC values exceeding 0.90 across both short-term and long-term prediction horizons. Notably, some models provided rolling predictions updated every few hours, facilitating real-time monitoring in high-risk ICU environments.

The selection of prediction windows varied widely across studies, reflecting different clinical goals. Approximately one-third of studies focused on near-term AKI risk prediction (eg, within 12 or 24 h), which aligns with the need for timely intervention. Others explored longer windows (eg, within 72 h or over the entire ICU stay), enabling broader treatment planning. While this diversity reflects clinical heterogeneity, it also complicates cross-study comparisons. Clear definitions and justifications for chosen time windows remain necessary to ensure reproducibility and clinical relevance. From a feature engineering perspective, dynamic models often leverage temporal trends or deltas (eg, creatinine change over 12 h, trajectory of urine output), which are more predictive than static values alone.

Despite their promise, dynamic models pose notable challenges. They are computationally intensive and require high-resolution, longitudinal data that may not be consistently available across institutions. Moreover, their complexity can hinder interpretability and limit clinical acceptance. Calibration metrics for these models were rarely reported, further raising concerns about their reliability in practice. In summary, dynamic prediction models represent a critical advancement in AKI risk stratification by aligning prediction capabilities with the temporal nature of ICU patient trajectories. However, to facilitate clinical adoption, future studies should prioritize model explainability, report comprehensive performance metrics, and develop user-friendly tools capable of integrating into real-time workflows.

#### External validation

External validation is essential for assessing whether a prediction model can generalize beyond its development dataset. While internal validation methods such as random sampling or cross-validation are useful for estimating in-sample performance, they often overestimate a model’s true utility in clinical practice.^11^

One-third of the included studies conducted external validation using independent datasets not involved in model development. Most of these external validations used publicly available databases, allowing for comparisons across studies.^88^ A few studies used institutional data from a different hospital or region, which more closely reflected real-world clinical deployment scenarios. The results from external validations were generally lower than those reported from internal evaluations. In studies comparing performance metrics directly, AUROC values often declined by nearly 0.05-0.1 when applied to external cohorts. This performance degradation was often found in models trained on single-center data with limited demographic or clinical diversity. Contributing factors might include differences in population characteristics, laboratory reference ranges, data coding standards, and clinical workflows.

Importantly, the absence of external validation in over two-thirds of the studies remains a significant limitation. Without robust out-of-sample testing, it is difficult to assess whether these models can reliably support clinical decisions in different patient populations or hospital systems. Models developed and validated on the same dataset may inadvertently reflect institution-specific patterns, limiting transportability. Moving forward, future studies should perform external validation, ideally across multiple centers and patient subgroups.

#### Interpretability

Interpretability remains a critical challenge in the clinical adoption of AI models for AKI prediction in ICU settings. While complex models, particularly DL architectures, often demonstrate superior predictive performance, their “black-box” nature can hinder clinician trust and limit real-world implementation. Among the 47 studies included in this review, 33 incorporated any form of interpretability analysis, and the depth and rigor of these methods varied substantially.

The most widely used interpretability technique was SHAP, which provides a model-agnostic, locally accurate explanation of feature contributions and is particularly well suited for complex models. Other reported methods included LIME, iBreakDown, Gini importance scores, permutation feature importance, and visualization of attention weights. A few studies employed custom scoring systems or tree-based logic, which provided inherently interpretable structures. Despite these advances, nearly half of the studies did not report any interpretability mechanism, leaving a substantial gap in transparency and limiting clinical integration. Even among those reporting feature importance, few translated these insights into clinically meaningful narratives or user-facing tools, such as dashboards or decision support interfaces.

Interpretability should not be viewed as an optional feature but as a core requirement for responsible AI in healthcare. Future studies should not only apply advanced interpretability methods but also prioritize the communication of model logic in formats accessible to clinical end users. Emerging techniques, such as natural language reasoning, counterfactual explanations, and integration with clinical knowledge graphs, offer promising pathways to enhance transparency.^87^ Furthermore, regulatory and ethical considerations increasingly demand that black-box models be accompanied by robust explanations, particularly in high-stake domains like critical care.^87^

### Data utilization

#### Predictive variables

Among the included 47 studies, the diversity of predictive variables highlights the complexity of AKI risk prediction. Frequently used laboratory parameters included serum creatinine, blood urea nitrogen, potassium, albumin, and platelet count. Frequently assessed vital signs encompassed heart rate, respiratory rate, and mean arterial pressure. These variables demonstrated utility in predicting AKI risk and monitoring dynamic changes in patient conditions.^89^^,^^90^ The influence of comorbidities, such as diabetes, chronic kidney disease, and congestive heart failure, on AKI risk was also investigated in the selected studies. These factors were consistently ranked among the top predictors due to their direct impact on renal function and patient prognosis. Moreover, interventions like mechanical ventilation and vasopressor use were also risk factors for AKI, as they might cause hemodynamic instability. Emerging biomarkers, such as NGAL, KIM-1, and L-FABP, showed potential in enhancing prediction models by providing early signals of kidney injury.^91–95^ However, the use of these biomarkers was limited due to cost and accessibility constraints. Despite these challenges, their integration into ML models have the potential to improve AKI prediction model performance.

#### Missing data handling

Handling missing data remains a notable limitation in current AKI prediction studies. Among the 47 included studies, most used simple strategies such as excluding variables with high data missing rates (typically >20%–40%) and applying mean or median imputation for the remaining data. Although straightforward, these methods assume that data are missing at random and may introduce bias, especially in ICU settings where missingness often reflects clinical decisions or disease severity.

A smaller group of studies adopted more advanced approaches, including multiple imputation by chained equations (MICE), RF-based imputation, and regression-based methods. Multiple imputation techniques, such as MICE, better account for uncertainty and preserve statistical properties compared to single-value imputation (eg, mean or median imputation), which can cause serious bias by underestimating variability.^96^ One study applied a generative adversarial imputation network, showing emerging interest in DL for this task.^47^ However, very few studies discussed the rationale behind their chosen methods, and none performed direct comparisons to assess the impact on model performance.

Future research should report missing data handling strategies more transparently, consider the possible causes of missingness, and evaluate how different imputation techniques affect model accuracy and clinical applicability.

### Risk of bias and clinical applicability

Despite notable progress in predictive modeling for AKI in ICU settings, most models remain far from clinical integration. The PROBAST assessment suggested a high risk of bias in most studies, especially in the analysis domain. Additionally, the TRIPOD evaluation indicated that although essential reporting elements were generally addressed, complete adherence to the full checklist was uncommon, indicating challenges in thorough and transparent reporting of prediction models. Among the 47 included studies, only 2 employed prospective data collection, and even fewer progressed to real-world implementation. One study trained an ML model on a prospectively collected cohort and externally validated it, providing an additional web-based calculator for clinical use.^34^ However, this tool has not been embedded into EHR systems or assessed in real-time workflows, underscoring its translational limitations. The other prospective study focused on risk factor analysis and LR-based prediction but similarly did not proceed to clinical deployment.^74^

In terms of model presentation, less than 15% of studies offered any usable interface, such as web calculators, nomograms, or simplified risk scores. The majority neither described clinical integration pathways nor addressed practical concerns like user testing, interpretability at the bedside, or embedding into clinical decision support systems. Without actionable outputs or clinician-facing tools, these models remain research artifacts rather than deployable interventions. Even among models with strong performance and external validation, few addressed the practical barriers to implementation. Critical steps such as workflow compatibility, clinician trust-building, and impact evaluation were almost absent. Furthermore, interpretability was underreported or superficially addressed in many studies, adding to the difficulty of clinical translation.

To narrow the gap between development and application, future research should focus not only on model accuracy but also on clinical usability. This includes involving stakeholders in design, evaluating feasibility for EHR integration, and conducting prospective impact studies in diverse healthcare settings. Ultimately, making AKI prediction models clinically actionable requires a paradigm shift, from performance-centric development toward implementation-aware design, with attention to usability, transparency, and integration in the complex ICU environment. To overcome these challenges, it is essential to facilitate interdisciplinary collaboration among computer scientists, clinicians, and policymakers. Combining expertise across fields can help address technical limitations and practical implementation barriers.^97^

### Limitations

This study has its limitations. First, although our search strategy covered multiple authoritative databases and employed a systematic approach, it is possible that some relevant studies were overlooked, particularly those preprints or non-English literature. Additionally, while the PROBAST provided valuable support for assessing bias and applicability, evaluating the risk of bias still involves a degree of subjectivity. Different researchers may prioritize varying aspects of bias, potentially introducing variability. Furthermore, this review could not perform a meta-analysis due to the high heterogeneity among the included studies, jeopardizing meaningful statistical aggregation and quantitative evaluation.

## Conclusions

This systematic review comprehensively evaluated the AI models for the prediction of AKI in the ICU. It provided a detailed overview of current AKI prediction studies on modeling methods, data utilization, and clinical applicability. Our findings highlight the advantages of AI models in handling complex ICU data, capturing temporal dynamics, and supporting early prediction of AKI. The real-time dynamic AKI prediction models help identify high-risk patients early and facilitate timely clinical interventions. However, limitations are still present in current studies, including inadequate generalizability and interpretability of models, low proportions of external validation, and barriers to data sharing due to technical and ethical challenges. These issues limit the broader adoption of prediction models in clinical practice. In the future, promoting cross-institutional data sharing, conducting multicenter studies, developing dynamic and interpretable prediction models, and making these models user-friendly will be key research directions. This review highlights the potential of AI models in AKI prediction and identifies critical challenges and areas for improvement, offering valuable insights for clinicians, researchers, and policymakers in critical care.

## Supplementary Material

ooaf065_Supplementary_Data

## Data Availability

No datasets were generated or analyzed during the current study. Data supporting the findings are publicly available in the manuscript of each study.
