# Supplementary material for: Artificial intelligence models for predicting acute kidney injury in the intensive care unit: a systematic review of modeling methods, data utilization, and clinical applicability
Source: JAMIA Open. 2025 Jul 3;8(4):ooaf065. doi: 10.1093/jamiaopen/ooaf065 (PMC12225755; doi:10.1093/jamiaopen/ooaf065)
Supplement: ooaf065_Supplementary_Data [file ooaf065_supplementary_data.zip › Revised Supplementary Materials 2.pdf]

Supplementary Materials of AI Models for Predicting Acute Kidney Injury in the ICU: A Systematic Review of Modeling Methods, Data Utilization, and Clinical Applicability

Tongyue Shi, Yu Lin, Huiying Zhao, and Guilan Kong*

Table S1: Search terms used across multiple databases.

Database	Search Terms	Number of Records Found
PubMed	(("intensive care units"[Mesh] OR "critical care"[Mesh] OR "critical illness"[Mesh] OR "ICU"[tiab] OR "intensive care unit"[tiab] OR "critical care"[tiab] OR "critical illness"[tiab]) AND ("Acute Kidney Injury"[Mesh] OR "acute kidney injury"[tiab] OR "AKI"[tiab] OR "acute renal injury"[tiab] OR "acute renal damage"[tiab] OR "acute renal failure"[tiab] OR "acute kidney failure"[tiab] OR "ARF"[tiab] OR "acute renal insufficiency"[tiab]) AND ("early diagnosis"[Mesh] OR "risk assessment"[Mesh] OR "early prediction"[tiab] OR "early identification"[tiab] OR "early detection"[tiab] OR "early onset"[tiab] OR "early warning"[tiab] OR "risk prediction"[tiab] OR "risk stratification"[tiab] OR "risk assessment"[tiab]) AND ("Artificial Intelligence"[Mesh] OR "Machine Learning"[Mesh] OR "Deep Learning"[Mesh] OR "Knowledge Graph"[tiab] OR "KG"[tiab] OR "Large Language Model"[tiab] OR "LLM"[tiab] OR "transformer"[tiab] OR "Data Mining"[tiab] OR "neural network"[tiab] OR "decision tree"[tiab] OR "random forest"[tiab] OR "support vector machine"[tiab] OR "SVM"[tiab] OR "Xgboost"[tiab] OR "adaboost"[tiab] OR "gradient boosting"[tiab] OR "naive bayes"[tiab] OR "regression model"[tiab] OR "natural language processing"[tiab] OR "NLP"[tiab] OR "temporal modeling"[tiab] OR "sequence modeling"[tiab]))	118
Scopus	(TITLE-ABS-KEY("ICU" OR "intensive care unit" OR "critical care" OR "critical illness")) AND (TITLE-ABS-KEY("acute kidney injury" OR "AKI" OR "acute renal injury" OR "acute renal damage" OR "acute renal failure" OR "acute kidney failure" OR "ARF" OR "acute renal insufficiency")) AND (TITLE-ABS-KEY("early prediction" OR "early identification" OR "early detection" OR "early onset" OR "early warning" OR "risk prediction" OR "risk stratification" OR "risk assessment")) AND (TITLE-ABS-KEY("artificial intelligence" OR "AI" OR "machine learning" OR "deep learning" OR "knowledge graph" OR "KG" OR "large language model" OR "LLM" OR "transformer" OR "data mining" OR "neural network" OR "decision tree" OR "random forest" OR "support vector machine" OR "SVM" OR "Xgboost" OR "adaboost" OR "gradient boosting" OR "naive bayes" OR "regression model" OR "natural language processing" OR "NLP" OR "temporal modeling" OR "sequence modeling"))	368
Web of Science	TS=("ICU" OR "intensive care unit" OR "critical care" OR "critical illness") AND TS=("acute kidney injury" OR "AKI" OR "acute renal injury" OR "acute renal damage" OR "acute renal failure" OR "acute kidney failure" OR "ARF" OR "acute renal insufficiency") AND TS=("early prediction" OR "early identification" OR "early detection" OR "early onset" OR "early warning" OR "risk prediction" OR "risk stratification" OR "risk assessment") AND TS=("artificial intelligence" OR "AI" OR "machine learning" OR "deep learning" OR "knowledge graph" OR "KG" OR "large language model" OR "LLM" OR "transformer" OR "data mining" OR "neural network" OR "decision tree" OR "random forest" OR "support vector machine" OR "SVM" OR "Xgboost" OR "adaboost" OR "gradient boosting" OR "naive bayes" OR "regression model" OR "natural language processing" OR "NLP" OR "temporal modeling" OR "sequence modeling")	267
Embase	('intensive care unit'/exp OR 'critical care'/exp OR 'critical illness'/exp OR ICU:ti,ab OR 'intensive care unit':ti,ab OR 'critical care':ti,ab OR 'critical illness':ti,ab) AND ('acute kidney injury'/exp OR 'AKI':ti,ab OR 'acute kidney injury':ti,ab OR 'acute renal injury':ti,ab OR 'acute renal damage':ti,ab OR 'acute renal failure':ti,ab OR 'acute kidney failure':ti,ab OR 'ARF':ti,ab OR 'acute renal insufficiency':ti,ab) AND ('early diagnosis'/exp OR 'risk assessment'/exp OR 'early prediction':ti,ab OR 'early identification':ti,ab OR 'early detection':ti,ab OR 'early onset':ti,ab OR 'early warning':ti,ab OR 'risk prediction':ti,ab OR 'risk stratification':ti,ab OR 'risk assessment':ti,ab) AND ('artificial intelligence'/exp OR 'machine learning'/exp OR 'deep learning'/exp OR 'knowledge graph':ti,ab OR KG:ti,ab OR 'large language model':ti,ab OR LLM:ti,ab OR transformer:ti,ab OR 'data mining':ti,ab OR 'neural network':ti,ab OR 'decision tree':ti,ab OR 'random forest':ti,ab OR 'support vector machine':ti,ab OR SVM:ti,ab OR Xgboost:ti,ab OR	494

	adaboost:ti,ab OR 'gradient boosting':ti,ab OR 'naive bayes':ti,ab OR 'regression model':ti,ab OR 'natural language processing':ti,ab OR NLP:ti,ab OR 'temporal modeling':ti,ab OR 'sequence modeling':ti,ab)	
IEEE Xplore	(("ICU" OR "intensive care unit" OR "critical care" OR "critical illness") AND ("acute kidney injury" OR "AKI" OR "acute renal injury" OR "acute renal damage" OR "acute renal failure" OR "acute kidney failure" OR "ARF" OR "acute renal insufficiency") AND ("early prediction" OR "early identification" OR "early detection" OR "early onset" OR "early warning" OR "risk prediction" OR "risk stratification") AND ("artificial intelligence" OR "AI" OR "machine learning" OR "deep learning" OR "knowledge graph" OR "KG" OR "large language model" OR "LLM" OR "transformer" OR "data mining" OR "neural network" OR "decision tree" OR "random forest" OR "support vector machine" OR "SVM" OR "Xgboost" OR "adaboost" OR "gradient boosting" OR "naive bayes" OR "regression model" OR "natural language processing" OR "NLP" OR "temporal modeling" OR "sequence modeling"))	16
ACM Digital Library	(("ICU" OR "intensive care unit" OR "critical care" OR "critical illness") AND ("acute kidney injury" OR "AKI" OR "acute renal injury" OR "acute renal damage" OR "acute renal failure" OR "acute kidney failure" OR "ARF" OR "acute renal insufficiency") AND ("early prediction" OR "early identification" OR "early detection" OR "early onset" OR "early warning" OR "risk prediction" OR "risk stratification") AND ("artificial intelligence" OR "AI" OR "machine learning" OR "deep learning" OR "knowledge graph" OR "KG" OR "large language model" OR "LLM" OR "transformer" OR "data mining" OR "neural network" OR "decision tree" OR "random forest" OR "support vector machine" OR "SVM" OR "Xgboost" OR "adaboost" OR "gradient boosting" OR "naive bayes" OR "regression model" OR "natural language processing" OR "NLP" OR "temporal modeling" OR "sequence modeling"))	42

Table S2. Summary of key characteristics in the selected studies.

Study	Country	Study design	Data source	Population	Research purpose	Sample size in the training dataset	Sample size in the validation dataset	AKI definition	Number of patients with AKI in the training dataset	Observed AKI incidence in the training dataset
Zhang et al., 2024[1]	China	Multicenter retrospective cohort study	eICU-CRD and MIMIC-III	ICU patients with rhabdomyolysis	To predict the occurrence of AKI in rhabdomyolysis patients	Not reported (nearly 70% of 938)	Not reported (nearly 30% of 938)	KDIGO	Not reported (nearly 70% of 666)	Not reported (nearly 71%)
Yang et al., 2024[2]	China	Multicenter retrospective cohort study	Development: MIMIC-IV; Validation: eICU-CRD and Southeast University Zhongda Hospital in China	ICU patients	To continuously predict AKI risk within 24 h for critically ill patients	17988	3175 (eICU-CRD), 3025 (Southeast University Zhongda Hospital)	KDIGO	6628	36.85%
Tu et al., 2024[3]	China	Single-center retrospective cohort study	MIMIC-IV	ICU patients with cirrhosis	To detect the risk of AKI occurring within 7 days after admission to the ICU in patients with cirrhosis	836	418	KDIGO	492	58.85%
Tan et al., 2024[4]	United States	Single-center retrospective cohort study	MIMIC-IV	ICU patients	To forecast AKI with a lead time of at least 12 hours ahead of clinical manifestation	38,400	10,586	KDIGO	14,168	36.90%
Sun et al., 2024[5]	China	Multicenter prospective cohort study	Jiefang campus and Binjiang campus of the Second Affiliated Hospital of Zhejiang University School of Medicine in China	ICU patients	To predict AKI in critically ill patients within 7 days	421	167	KDIGO	117	27.79%
Shi et al., 2024[6]	China	Single-center retrospective cohort study	MIMIC-IV	ICU patients with sepsis	To predict the occurrence of AKI in patients diagnosed with sepsis	10,575	Not reported	KDIGO	8,575	81.09%
Lyu et al., 2024[7]	Switzerland	Multicenter retrospective cohort study	HiRID-II and MIMIC-IV	ICU patients	Early prediction of AKI in ICU settings	Not reported	Not reported	KDIGO	Not reported	Nearly 50%
Lu et al., 2024[8]	China	Single-center retrospective cohort study	The Central Hospital of Lishui City,	ICU patients with cerebral infarction	To predict cerebral infarction patients with AKI	3098	822	KDIGO	1355	43.74%

			Zhejiang Province in China						
Liu et al., 2024[9]	China	Single-center retrospective cohort study	Guangdong Provincial People's Hospital (Guangdong Academy of Medical Sciences), Southern Medical University	Patients in the intensive care unit of cardiac surgery (CS-ICU)	To predict severe AKI after total aortic arch replacement for acute type A aortic dissection	394	178	KDIGO	93	23.60%
Lin et al., 2024[10]	China	Single-center retrospective cohort study	MIMIC-IV	ICU patients with acute pancreatitis	To predict the incidence of AKI within seven days following the patient's admission to the ICU	865	370	KDIGO	467	53.99%
Li et al., 2024[11]	China	Multicenter retrospective cohort study	MIMIC-IV and Xiangya Hospital of Central South University in China	Critically Ill Elderly Patients	To predict the risk and prognosis of AKD in the elderly	3542	280	KDIGO	2661	75.13%
Zheng et al., 2023[12]	China	Multicenter retrospective cohort study	eICU-CRD and MIMIC-III	Critically ill cirrhotic patients	To predict the development of AKI in critically ill cirrhotic patients	804	789	KDIGO	212	26.37%
Zhang et al., 2023[13]	China	Single-center retrospective cohort study	MIMIC-III	ICU patients	AKI prediction at 3 prediction windows, where data imputation approaches were evaluated accordingly	21,772	Not reported	KDIGO	8695	39.94%
Wu et al., 2023[14]	China	Single-center retrospective cohort study	The Dongyang People's Hospital in China	NICU patients	To predict AKI following brain surgery from patients admitted to the NICU	408	174	KDIGO	Not reported (121 for all cohorts)	Not reported (20.79% for all cohorts)
Wu et al., 2023[15]	Germany	Single-center retrospective cohort study	eICU-CRD	ICU patients	Early detection of acute kidney failure	403478 (all prediction samples) * 0.8	403478 (all prediction samples) * 0.1	Diagnosis flowchart for renal system provided by clinicians at German Heart Center Munich and ICD code 584.9 (Acute renal failure, unspecified)	70289 (all prediction samples with AKI) * 0.8	Not reported (Nearly 17.4%)

Shi et al., 2023[16]	China	Multicenter retrospective cohort study	eICU-CRD and MIMIC-IV	ICU patients with gastrointestinal bleeding	AKI prediction in patients with gastrointestinal bleeding admitted to the ICU	6,679	5,832	KDIGO	5679	85.03%
Persson et al., 2023[17]	Sweden	Single-center retrospective cohort study	MIMIC-IV	ICU patients	Hourly predictions of AKI	9,996	7,797	KDIGO	2199	22.00%
Peng et al., 2023[18]	China	Multicenter retrospective cohort study	eICU-CRD and MIMIC-IV	ICU patients with traumatic brain injury	To predict of Severe Acute Kidney Injury Following Traumatic Brain Injury	808	524	KDIGO	60	7.43%
Pan et al., 2023[19]	China	Single-center retrospective cohort study	MIMIC-III	ICU patients	To predict new-onset AKI 3–6–9–12 h ahead	2,260 (all)	Not reported	KDIGO	1130 (all)	50.00%
Luo et al., 2023[20]	China	Single-center retrospective cohort study	MIMIC-III	ICU patients	Early Prediction Model for Acute Kidney Injury within 72-Hours Post-ICU Admission	15,218 (all)	Not reported	KDIGO	4902 (all)	32.21%
Jiang et al., 2023[21]	China	Single-center retrospective cohort study	MIMIC-IV	ICU patients with acute pancreatitis	To construct and validate a risk assessment model for acute kidney injury (AKI) in patients with acute pancreatitis (AP) in the intensive care unit (ICU).	674	289	KDIGO	491 (all)	Nearly 50.99%
Huang et al., 2023[22]	China	Multicenter retrospective cohort study	MIMIC-IV and eICU-CRD	ICU patients	To detect the onset of severe AKI in the first week of intensive care unit (ICU) stay during the initiation of MV	3,986	2,704	KDIGO	421	10.56%
Begum et al., 2023[23]	India	Single-center retrospective cohort study	MIMIC-III	ICU patients	Early detection of acute kidney disease	28,516 (all)	Not reported	KDIGO	Not reported	Not reported
Yue et al., 2022[24]	China	Single-center retrospective cohort study	MIMIC-III	ICU patients with sepsis	To establish and validate predictive models based on novel machine learning (ML) algorithms for AKI in critically ill patients with sepsis.	3,176 (all)	Not reported	KDIGO	2397 (all)	75.74%
Zhang et al., 2022[25]	China	Multicenter retrospective cohort study	MIMIC-IV, eICU-CRD and Zigong Fourth People's Hospital from China	ICU patients with sepsis	To develop an accurate ensemble model to predict the risk of S-AKI based on easily available clinical information	21,038	25,037	KDIGO	15785	75.03%
Zeng et al., 2022[26]	China	Multicenter retrospective cohort study	MIMIC-III and MIMIC-IV	ICU patients with sepsis	To predict 2- and 7-day septic AKI	10,734	7,413	KDIGO	3876	36.11%
Zhang et al., 2022[27]	China	Multicenter retrospective cohort study	MIMIC-III and a tertiary teaching hospital in China	ICU patients with acute cerebrovascular disease	To predict the risk of AKI through machine learning methods in critical care patients with acute cerebrovascular disease.	1,467	734	KDIGO	268	18.27%
Wen et al., 2022[28]	China	Single-center retrospective cohort study	MIMIC-III	ICU patients	To predict risk factors for AKI in critically ill patients within the first 7 days after admission to the intensive care unit (ICU).	561 (all)	Not reported	KDIGO	440 (all)	78.43%

Vagliano et al., 2022[29]	The Netherlands	Single-center retrospective cohort study	MIMIC-III	ICU patients	To predict AKI that includes not only clinical variables, but also clinical notes and medical terminologies.	37,707	47,10	KDIGO	11170	29.62%
Gao et al., 2022[30]	China	Single-center retrospective cohort study	MIMIC-III	ICU patients	To predict AKI occurrence in ICU patients and identify key predictive features.	30,020 (all)	Not reported	KDIGO	17222 (all)	57.40%
Begum et al., 2022[31]	India	Single-center retrospective cohort study	MIMIC-III	ICU patients	To develop and validate a machine learning model with novel kidney biomarkers for predicting AKI onset and progression in ICU patients.	Not reported	Not reported	KDIGO	Not reported	Not reported
Cai et al., 2022[32]	China	Multicenter retrospective cohort study	MIMIC-III and MIMIC-IV	ICU patients with acute myocardial infarction	Develop and validate machine learning models to predict the risk of AKI in acute myocardial infarction (AMI) patients.	2,624	1,258	KDIGO	773	29.46%
Qian et al., 2021[33]	China	Single-center retrospective cohort study	MIMIC-III	ICU patients	To compare the performance of different machine learning and deep learning models in predicting AKI within 72 hours of ICU admission.	7,858 (all)	Not reported	KDIGO	3929 (all)	50.00%
Luo et al., 2021[34]	China	Single-center retrospective cohort study	MIMIC-III	ICU patients with sepsis	To use machine learning methods to predict and distinguish between transient and persistent sepsis-associated AKI (SA-AKI) in critically ill patients.	4,188	1,796	Acute Disease Quality Initiative (ADQI)	2,611	62.34%
Le et al., 2021[35]	United States	Single-center retrospective cohort study	MIMIC-III	ICU patients	To develop a convolutional neural network (CNN)-based model for predicting AKI up to 48 hours before onset in ICU patients, using structured EHR data and unstructured clinical notes.	Approximately 90% of the dataset (around 11,112 patient encounters)	Approximately 10% of the dataset (around 1,235 patient encounters)	KDIGO	857	7.70%
Gong et al., 2021[36]	China	Single-center retrospective cohort study	MIMIC-III	ICU patients	To develop a machine-learning framework for predicting and interpreting AKI risk in ICU patients within 72 hours of admission, addressing both prediction accuracy and interpretability.	Approximately 80% of the dataset (around 9,957 patients)	Approximately 20% of the dataset (around 2,490 patients)	KDIGO	1739	Nearly 14%
Chiorean et al., 2021[37]	United States	Single-center retrospective cohort study	MIMIC-III	ICU patients	To develop a reproducible ETL (Extraction, Transformation, and Loading) pipeline for ICU data, enabling window-based AKI prediction	26,171	Not reported	KDIGO	7337	28.03%
Alfieri et al., 2021[38]	Italy	Multicenter retrospective cohort study	eICU and MIMIC-III	ICU patients	To develop and evaluate a deep-learning model to continuously predict severe AKI (stages 2/3) in ICU patients based on urine output trends, comparing its performance with a logistic regression model.	21,681	3,331	AKIN	658	3.03%
Wang et al., 2020[39]	China	Multicenter retrospective cohort study	ICUC dataset from West China hospital in China and MIMIC-III	ICU patients	To develop and evaluate an Ensemble Time Series Model (ETSM) for early AKI prediction using vital signs, laboratory results, and medication data to address the	11,501 samples for 24-hour prediction, 10,921 samples for 48-hour prediction	46,593 samples for 24-hour prediction, 30,217	KDIGO	2,035	16.00%

					challenges of imbalanced and sparse datasets.		samples for 48-hour prediction			
Rank et al., 2020[40]	Germany	Single-center retrospective cohort study	Tertiary care center for cardiovascular diseases, Germany	Cardiothoracic ICU patients	To develop a recurrent neural network (RNN) model for real-time prediction of AKI following cardiothoracic surgery and compare its performance to that of experienced clinicians.	2,180	350	KDIGO	1308	60.00%
Matsuura et al., 2020[41]	Japan	Multicenter retrospective cohort study	21 ICUs in Japanese hospitals	ICU patients	To develop and validate a concise scoring method, Persistent AKI Risk Index (PARI), for identifying ICU patients at high or low risk of persistent AKI.	4,151	4,169	KDIGO	537	12.94%
Zimmerman et al., 2019[42]	United States	Single-center retrospective cohort study	MIMIC-III	ICU patients	To develop and validate a predictive model for AKI among ICU patients using multivariate physiological measurements from the first 24 hours of ICU admission.	23950 (all)	Not reported	KDIGO	3945 (all)	16.47%
Wang et al., 2019[43]	China	Single-center retrospective cohort study	West China Critical Care Information System in China	ICU patients	To develop a predictive model for AKI using the XGBoost-VM algorithm, incorporating vital signs and medication information to forecast AKI occurrence 24 and 48 hours ahead.	11,501 (24-hour prediction),10,921 (48-hour prediction)	Not reported	KDIGO	1,490 (24-hour prediction), 910 (48-hour prediction)	12.96% (24-hour prediction), 8.33% (48-hour prediction)
Parreco et al., 2019[44]	United States	Single-center retrospective cohort study	eICU-CRD	ICU patients	To compare the performance of machine learning algorithms in predicting AKI in ICU patients using trends in laboratory measurements and vital signs.	151,098 (all)	Not reported	KDIGO	8408 (all)	5.56%
Sun et al., 2019[45]	United States	Single-center retrospective cohort study	MIMIC-III	ICU patients	To develop predictive models for early identification of AKI risk in ICU patients using both structured physiological measurements and unstructured clinical notes within 72 hours of ICU admission.	11,558	5,000	KDIGO	1,927	16.67%
Chiofolo et al., 2019[46]	United States	Single-center retrospective cohort study	Mayo Clinic in Rochester, Minnesota in USA	ICU patients	To develop and validate a random forest-based prediction model for continuous AKI risk monitoring during ICU admission.	4,572	1,958	AKIN	1355	29.64%
Mao et al., 2017[47]	China	Single-center prospective observational study	Shanghai 9th People's Hospital in China	ICU patients	To evaluate the risk factors associated with acute kidney injury (AKI) and dialysis among ICU patients.	1,544	Not reported	AKIN	748	48.45%

Abbreviations: AKI: Acute Kidney Injury; eICU-CRD: eICU Collaborative Research Database; MIMIC: Medical Information Mart for Intensive Care; KDIGO: Kidney Disease: Improving Global Outcomes; ICU: Intensive Care Unit; NICU: Neurological Intensive Care Unit; AKIN: Acute Kidney Injury Network; ADQI: Acute Disease Quality Initiative; CNN: Convolutional Neural Network; SA-AKI: Sepsis-Associated Acute Kidney Injury; TAAR: Total Arch Replacement; ATAAD: Acute Type A Aortic Dissection; GBT: Gradient Boosted Trees; ETSM: Ensemble Time Series Model; ETL: Extraction, Transformation, and Loading; PARI: Persistent AKI Risk Index; XGBoost: Extreme Gradient Boosting; VM: Variance Mitigated; ICD: International Classification of Diseases; S-AKI: Sepsis Acute Kidney Injury; AMI: Acute Myocardial Infarction; CS-ICU: Cardiac Surgery Intensive Care Unit.

Table S3. Characteristics of predictive features in the selected studies.

Study	Feature selection method	Predictor count	Predictive features	Missing Data Handling
Zhang et al., 2024[1]	Not reported	28	Age, Male, MAP, Temperature, Heart rate, S_PO_2, Respiratory rate, CK, Creatinine, BUN, ALT, AST, Albumin, Bicarbonate, Hematocrit, Hemoglobin, Calcium, Sodium, Potassium, Lactate, RDW, Phosphate, Atrial fibrillation, Acute hepatic failure, Stroke, Acute myocardial infarction, SOFA, APS III	Variables with > 40% missing excluded; median imputation for remaining
Yang et al., 2024[2]	Not reported	152	Demographics: Age, Gender, Weight, Height, Chronic kidney disease, Diabetes, Chronic pulmonary disease, Congestive heart failure, Moderate/Severe liver disease, Current ICU length of stay; Vital signs: Heart rate, Temperature, Systolic blood pressure, Mean arterial pressure, Diastolic blood pressure, Respiration rate, SpO2; Laboratory values: pH, pO2, pCO2, FiO2, BaseExcess, Lactate, Glucose, Hematocrit, Hemoglobin, White blood cells, Platelet, Albumin, Aniongap, Bicarbonate, Blood urea nitrogen, Creatinine, Calcium, Chloride, Sodium, Potassium, International normalized ratio, Prothrombin time, Alanine aminotransferase, Alkaline phosphatase, Aspartate aminotransferase, Total bilirubin, Total 12-h urine output; Empiric features: PO2/FiO2 ratio, Blood Urea Nitrogen (BUN)/SCr ratio, Body Mass Index (BMI), lowest SCr value in the last 48 h, total-12 h-urine output/weight/12 h (UO_12h_Rt); Informative missingness features: Binary indicator to distinguish between the missing value and an actual clinical event of each vital sign and laboratory values; Trend features: The difference between the current record and the previous value of each vital sign and laboratory values; Statistics features: For vital signs, statistics (maximum, minimum, median, standard deviation, and differential SD) in a 24-h sliding window were counted	Forward the earlier available observation for imputation. (If there was no available observation, they used the median values for the training data to fill in the remaining missing values.)
Tu et al., 2024[3]	LASSO regression	8	MAP、WBC、TB、GCS、Cr、Alb、HR and PLT	Variables with > 20% missing excluded; continuous variables were imputed using mean (normal distribution) or median (skewed distribution); binary variables had no missing values
Tan et al., 2024[4]	Not reported	50	Not listed all	Median imputation for hourly data gaps; normalization applied
Sun et al., 2024[5]	Univariate logistic regression, gini importance, LASSO regression, and ridge regression	9	NGAL, IGFBP7, sCysC, CAF22, KIM-1, NT-proBNP, IL-6, IL-18, and L-FABP	Variables with > 30% missing excluded; median and random imputation
Shi et al., 2024[6]	Not reported	47	Demographic features: Age, gender, race; Characteristics of hospitalization: Admission type, first care unit; Vital signs: Temperature, HR, RR, SBP, DBP, MAP, SpO2; Laboratory parameters: pH, PaO2, PaCO2, HCO3, base excess, K, Na, Cl, Mg, Ca, PO4, WBC, RBC, PLT, HGB, HCT, glucose, BUN, Cr, PT, INR, PTT, urine volume; Comorbidities: Myocardial infarct, CHF, cerebrovascular disease, diabetes, peptic ulcer disease, severe liver disease, cancer, AIDS, CAD, AFib, ARDS; Rating scales: OASIS	Variables with > 20% missing excluded; median imputation
Lyu et al., 2024[7]	SHAP-based pre-filtering	23	Demographics: Age, gender; Vital signs: HR, SBP, DBP, MAP, RR, temperature; Laboratory values: Cr, BUN, Cl, K; Medications: Diuretics, antibiotics; Procedures: MV, RRT; Other: Time since admission	Partial imputation with forward filling for time series gaps

	combined with greedy forward selection.			
Lu et al., 2024[8]	LASSO, Boruta, mRMR, Relief, and XGBoost	14	Drinking, RR, MV, pulmonary infection, hemiplegia, diabetes, hypertension, NIHSS score, total cholesterol, LDL, BUN, Cr, K, GFR	Variables with >30% missing values were excluded; multiple imputation was used for missing values
Liu et al., 2024[9]	RF, SVM-RFE and Lasso regression	8	sCysC, APACHE II, Post_PCT, Post_AST, Post_LDH, uNAG, Pre_Cr and Post_PLT	Variables with incomplete data were excluded; normalization was applied for continuous variables
Lin et al., 2024[10]	RFE	51	Admission_type, admission_age, race, insurance, glucose_mean, HR_mean, SBP_mean, DBP_mean, MAP_mean, RR_mean, temperature_mean, SpO_2_mean, HCT_max, HGB_max, PLT_max, WBC_max, aniongap_min, BUN_max, calcium_max, chloride_max, Cr_max, glucose_max, sodium_max, K_max, INR_max, PT_max, PTT_max, ALT_max, ALP_max, AST_max, bilirubin_total_max, CHF, peripheral_vascular_disease, dementia, cerebrovascular_disease, CPD, peptic_ulcer_disease, mild_liver_disease, diabetes_without_cc, paraplegia, malignant_cancer, severe_liver_disease, metastatic_solid_tumor, AIDS, sepsis3, MV, RRT, vaso, UO	Variables with > 20% missing values excluded; multiple imputations applied for remaining missing data
Li et al., 2024[11]	Not reported	33	Age, gender, AKI stage; sepsis, hypertension, diabetes, CKD, CPD, CLD; MV, RRT, vasopressors; HR, RR, SBP, DBP; WBC, RBC, HGB, HCT, K, calcium, anion gap, PaO2, PaCO2, pH, glucose, BUN, Cr. These indicators were measured on day 1 of AKI diagnosis. BUN and Cr levels on day 3 were also obtained, along with corresponding delta BUN and delta Cr values on day 3 minus day 1.	Variables with >35% missing were excluded; multiple imputation used for remaining data
Zheng et al., 2023[12]	LASSO regression	8	sCr, total bilirubin, Mg, shock index, PT, and mean corpuscular hemoglobin	Individuals with any missing values were excluded
Zhang et al., 2023[13]	Not reported	25	ALB, APTT, arterial pH, bicarbonate, bilirubin, CKMB, creatinine, glucose, heart rate, hematocrit, hemoglobin, lactate, MBP, PCO2, PO2, RBC, respiratory rate, urine specific gravity, SpO2, temperature, urea, WBC, input fluid, urine output, ventilation status	Gaussian regression-based imputation for time-series data
Wu et al., 2023[14]	Backward feature selection, random forest sampling, and tenfold cross-validation	10	SaO2, Oxyhemoglobin saturation; OP_SBP_avg, average SBP; OP_DBP_min, minimum DBP; OP_DBP_max, maximum DBP; OP_SBP_min, minimum SBP	Variables with > 20% missing data were excluded; multiple imputation was applied for other missing data
Wu et al., 2023[15]	Two-sample z-test and SHAP-based feature importance	30	Patient Information (gender, age, admission weight, admission height), Vital Sign (heart rate, oxygen saturation, respiration, central venous pressure, systolic/mean/diastolic blood pressures), Intake Output (urine), Lab Test (GLC, K, HCO3, Na, Urea, Cr, Cl, Ca, GHBG, PLT, RBC, WBC, MCHC, MCV, MCH, RDW, Mg, MPV, PH, PAO2, PACO, Baseexcess, SAO2, Caionized, METHB, COHB)	Compared zero-imputation and mean-imputation; models trained with zero-imputed data showed better performance
Shi et al., 2023[16]	XGBoost-based feature importance	7	BIL_max, HCO3_min, BIL_min, RRT, MV, MAP, CKD	Not reported

Persson et al., 2023[17]	Collaboration with medical professionals	22	Age, Sex, HR, RR, BT, SBP, DBP, VP, PH, GLU, LAC, SCr, BIL, BUN, WBC, PLT, SpO2, FiO2, PaO2, INR, GCS, UO. Hourly values were used, and a last observation carried forward approach was used for any hours with missing information. For any hours with more than one measurement, hourly averages were used. Feature engineering was performed to obtain 2 additional variables: the creatinine ratio (ratio of the current value of creatinine to the minimum creatinine value during the last 7 days) and the creatinine difference (difference between the current value of creatinine and the minimum creatinine value during the last 2 days)	Last observation carried forward approach for hourly data; exclusion of variables with missing >20%; variables with missing <20% were imputed using median values
Peng et al., 2023[18]	RFE	15	Creatinine, UO, APTT, BUN, SOFA, RBC, Bicarbonate, APSIII, Chloride, RDW, GCS, Renal disease, Diabetes, CHF	Multiple imputation for variables with missing data
Pan et al., 2023[19]	Interaction and multicollinearity tests in multivariate models	19	Sex, Height, Weight, BMI, Age, Body temperature, HR, RR, SBP, DBP, SpO2, WBC, Hemoglobin, Hematocrit, PLT, Creatinine, BUN, Bicarbonate, Potassium	Linear interpolation for dynamic features; mean imputation for static features
Luo et al., 2023[20]	Recursive Feature Elimination with Cross-Validation (RFECV)	8	Frequency, TVU, Scr_baseline, BUN, K, PO2, GCS, PH	Generative adversarial imputation networks for missing data
Jiang et al., 2023[21]	LASSO regression	11	Weight, Sepsis, CHF, SOFA, Wbc max, Alb, Vasopressin, CCI, RR max, RR min, UO	Variables with > 20% missing data excluded; other missing values imputed using the MICE package
Huang et al., 2023[22]	Univariate and multivariate analyses	12 (AKI-23) and 8 (AKI-3)	Highest heart rate, Lowest diastolic blood pressure, Lowest systolic blood pressure, Highest FIO2 of ventilator, Haemoglobin level, Haematocrit level, Platelet count, Serum urea nitrogen level, Phosphorus level, Magnesium level, SCr level, Baseline SCr level	Baseline serum creatinine imputed when missing
Begum et al., 2023[23]	Not reported	33	Age, gender, Diastolic blood pressure, mean blood pressure, respiratory rate, spo2, body temperature, hemoglobin, Lab tests based on timestamps, Diabetes, hypertension, heart failure, chronic kidney disease, ICDcodes, Bilirubin, albumin, calcium, bicarbonate, glucose, potassium, platelet, sodium, BUN, eGFR, WBC, SCr, NSAIDs, sedative, vasopressor, mechanical ventilation	Pattern mixture model used for imputing non-random missing data
Yue et al., 2022[24]	Not reported	30	(1) demographic features, including sex, age, and ethnicity; (2) comorbidities, including congestive heart failure, hypertension, chronic pulmonary, diabetes, and liver disease; (3) vital signs, including heart rate, temperature, oxygen saturation (SpO2), systolic blood pressure (SysBP), and diastolic blood pressure (DiasBP); (4) laboratory parameters, including total bilirubin, anion gap, albumin, chloride, potassium, sodium, lactate, partial thromboplastin time (PTT), prothrombin time (PT), international normalized ratio (INR), creatinine, blood urea nitrogen (BUN), and glucose; (5) therapeutic and clinical managements, including mechanical ventilation and vasopressor use. For some variables with multiple measurements, we included the maximum and minimum values for analysis	Variables with > 20% missing values excluded; multiple imputation was applied to other variables
Zhang et al., 2022[25]	RFE	17	Age, anion gap, creatinine, hemoglobin, mean cell hemoglobin concentration (MCHC), phosphate, international normalized ratio (INR), platelet, total bilirubin, potassium, pH, lactate, PaO2, PaCO2, heart rate, temperature and SpO2	Zero-imputation for dynamic features and mean-imputation for static features

Zeng et al., 2022[26]	Not reported	25	Age, Female, LOS, Creatinine serum, Creatinine urine, PCR, Osmolality urine, Chloride, Albumin, Sodium, White blood cells, pH, Urea nitrogen, Potassium urine, Uric acid, Hemoglobin, CRP, Gentamicin, Hematocrit, Lactate, Lactate dehydrogenase, Base excess, NTproBNP, Ketone, Vancomycin	Zero-imputation with indicator for missing values
Zhang et al., 2022[27]	Not reported	23	Sex, Age, Length of stay in ICU, Hypertension, Diabetes, CRRT, Mechanical ventilation, Mannitol, Colloid bolus, NSAIDS, Diuretics, Vasoactive drugs, Aminoglycosides, Infection, White blood cell count, Hemoglobin, Platelet count, Creatinine, Blood urea nitrogen, Potassium, Sodium, Bicarbonate, Albumin	Patients with > 20% missing data were excluded; multiple imputation was performed for other variables
Wen et al., 2022[28]	LASSO regression	10	Hypertension, CAD, Cardiopulmonary bypass, CABG, SAPS II, CVPfirst, MAPmin, MAPmax	Random forest method was used for missing data imputation
Vagliano et al., 2022[29]	Not reported	27	Age, Gender, Ethnicity, Duration by ICU admission, Admission type, Vasopressors, Ventilation, Sedatives, Creatinine, Urea nitrogen 6hr, Urea nitrogen 12hr, Urea nitrogen 24hr, Sodium, Potassium, Chloride, Anion gap, Bicarbonate, Blood urea nitrogen, Glucose, Hematocrit, Hemoglobin, White blood cell count, Diastolic BP, Systolic BP, Heart rate, Respiratory rate, SpO2	Not reported
Gao et al., 2022[30]	SHAP analysis and redundant feature removal based on importance ranking	57	Demographics: age; gender; Vital Signs: HR; systolic/diastolic BP; body temperature; SpO2; Laboratory Values: SCr; BUN; glucose; albumin; bicarbonate; WBC count; potassium; chloride; Critical Illness Scores: SOFA; SAPS II; APS III; Interventions: mechanical ventilation; RRT; Comorbidities: sepsis; weight; height	Patients with > 20% missing data were excluded; multiple imputation was performed for the remaining variables
Begum et al., 2022[31]	Selection based on prior research and biomarker relevance	18	Novel Biomarkers: cystatin C; NGAL; KIM-1; IL-18; L-FABP; IGFBP-7; Traditional Clinical Features: age; gender; ethnicity; Vital signs: HR; RR; BP; SpO2; Lab values: SCr; urine output; BUN; HGB; Mechanical ventilation status.	Carry-forward imputation for missing values
Cai et al., 2022[32]	Iterative modeling with top 5, 10, 15, 20, and all variables	37	Comorbidities include AF; HF; DM; hypercholesterolemia; hypertriglyceridemia; hypertension; respiratory failure; VT; cardiogenic shock; Vital signs: first recorded results at the time of hospitalization, including HR; RR; body temperature; SBP; DBP; MAP; Laboratory parameters: first time after admission, including RBC; WBC; PLT; HGB; glucose; HCT; BUN; SCr; potassium; sodium; chloride; calcium; phosphorus; magnesium; bicarbonate; APTT; PT; INR; CK-MB; TNT	Multiple imputation for variables with <5% missing data; exclusion for variables with >5% missing data
Qian et al., 2021[33]	Not reported	17	Demographics: age; sex; BMI; Physiological Measures: maximum and minimum values of BUN; SCr; potassium; bicarbonate; HGB; calcium; glucose; INR; PT; PTT; WBC; PLT; SpO2; Urine Output: volume measurements over 24 hours	Multiple imputation using the MICE package
Luo et al., 2021[34]	Feature importance from XGBoost and LASSO refinement	14	Predictive Features: Demographics: age; Comorbidities: diabetes mellitus; CHF; CKD; Vital Signs: minimum PaO2; maximum PaCO2; MV; Laboratory Data: maximum anion gap; maximum lactate; maximum INR; maximum PTT; Other Factors: AKI stage by SCr and urine output criteria; RRT initiation	Random forest imputation for variables with ≤ 30% missing values
Le et al., 2021[35]	Not reported	8 structured features + Doc2Vec embeddings	Structured Data: age; DBP; SBP; body temperature; RR; HR; SpO2; GCS; Unstructured Data: clinical notes embedded using Doc2Vec	Last observation carried forward; remaining missing values imputed with median

Gong et al., 2021[36]	Through extensive literature review and consultation with health professionals	54	Demographics: age; gender; ethnicity; Vital signs: BMI; DBP; SBP; MAP; body temperature; HR; RR; SpO2; Laboratory measurements: ALB; bicarbonate; bilirubin; BUN; calcium; chloride; eGFR; glucose; HCT; HGB; INR; lactate; PLT; potassium; phosphate; PT; PTT; SCr; sodium; WBC; Comorbidities: diabetes; hypertension; hypotension; sepsis; other infection; CKD; CLD; chronic lung disease; CHF; anemia; obstructive uropathy; Medications, Procedures and Intravenous fluids: diuretics; vancomycin; other antibiotics; NSAIDs; ACEI/ARB; calcineurin inhibitors; mechanical ventilation; contrast; total volume of intravenous NS; RL; all crystalloids; all colloids within the first 24 h in ICU.	Multivariate imputation by chained equations (MICE) for < 40% missing data; exclusion of variables with > 40% missing
Chiorean et al., 2021[37]	Not reported	36	Demographics: age; gender; ethnicity; Comorbidities: CHF; peripheral vascular disease; hypertension; diabetes; liver disease; MI; CAD; cirrhosis; jaundice; Medications: diuretics; NSAIDs; radiocontrast agents; angiotensin receptor blockers; Lab-events: BUN; calcium; chloride; SCr; HGB; INR; PLT; potassium; PT; PTT; WBC; Chart-events: MAP; HR; SBP; DBP; RR; temperature; SpO2; glucose.	Mean imputation for the missing values
Alfieri et al., 2021[38]	Not reported	Not explicitly mentioned	Urine output trends normalized to ideal body weight (mL/kg/h); Features derived from sliding window analysis: minimum averages for each window size	Missing urine output values (if gap <9 hours) were distributed equally over the missing interval; creatinine values dragged forward if gap < 4 days
Wang et al., 2020[39]	Explicit indicators derived from vital signs and lab results; implicit indicators modeled via IFICF (Indicator Frequency and Inverse Cohort Frequency)	Not explicitly mentioned	Explicit Indicators: Vital signs (e.g., blood pressure, heart rate) and laboratory results (e.g., creatinine, potassium, hemoglobin); Implicit Indicators: Medication combinations represented as time series features	Missing values were filled with adjacent timestamp data; for features entirely missing, the median value was used
Rank et al., 2020[40]	Availability and clinical relevance	96	Static features: Age; sex; height; weight; surgery characteristics; Dynamic features: Laboratory results: SCr; urea; GFR; HGB; PLT; etc.; Vital signs: BP; HR; SpO2; Arterial blood gas values: pH; HCO3; lactate; Fluid output: Urine flow rate; Nephrotoxic agents: Binary variables for 22 medications	Except for the nephrotoxic agents, missing values were filled by forward imputation
Matsuura et al., 2020[41]	Univariable logistic regression for serum creatinine changes and multivariable regression for clinical risk factors	4	ΔCr: Changes between ICU admission and the following day (ΔCr ≥ 0.2 mg/dL, ΔCr ≥ 0.3 mg/dL, ΔCr ≥ 0.4 mg/dL); Risk Factors: Hyperbilirubinemia (≥2 mg/dL), Sepsis, Ventilator and/or vasopressor use	Not reported
Zimmerman et al., 2019[42]	Backward selection based	20	Demographics: Age, gender, ethnicity; Vital Signs: HR, SBP, DBP, SpO2; Laboratory Values: SCr, K, HGB, PLT, HCO3, Ca, BUN; Procedures: Mechanical ventilation status	Variables with > 20% missing excluded; MICE for variables with missing data

	on statistical significance			
Wang et al., 2019[43]	Not reported	Not explicitly mentioned	Vital Signs: Includes vital sign values (start/end timestamps) for HR, blood pressure, and others; Medication Information: Multi-dimensional vectorized drug combinations using TF-IDF	Missing values filled using the most recent values or the median of the cohort for fully missing timestamps
Parreco et al., 2019[44]	Least-squares fit for trends in lab and vital sign data	Not explicitly mentioned	Slope and daily values for minimum and maximum creatinine, BUN, Glucose, potassium, sodium, chloride, other serum laboratory values and vital signs	Not reported
Sun et al., 2019[45]	Univariate analysis, L2-regularization, and term frequency-inverse document frequency (TF-IDF) for text data	Not explicitly mentioned	Patient demographics, clinical notes during the first day of ICU admission and 72-hour serum creatinine after admission, Bag-of-words features from clinical notes, including terms like "lasix," "insulin," and "cardiac output"	Variables with >20% missing data excluded; MICE used for <20% missing data
Chiofolo et al., 2019[46]	Univariate AUC > 0.5, dimensionality reduction, and clinical judgment	19	BUN, pH, Age, noninvasive DBP, Temperature, noninvasive MAP, HCT, Sodium, Potassium, estimated GFR based on SCr, median UO at 12 and 24 hours, shock index based on noninvasive and invasive DBP, pulse pressure, delivered tidal volume, PaO2/FiO2, net fluid balance, and cumulative dose of normal saline.	Not reported
Mao et al., 2017[47]	Not reported	9	Demographic Data: Age, Gender; Clinical Information: Mechanical ventilation, Vasopressors, SOFA, APACHE-II; Laboratory Parameters: SCr, Urea	Not reported

Abbreviations: RFE: Recursive Feature Elimination; LASSO: Least Absolute Shrinkage and Selection Operator; SHAP: Shapley Additive Explanations; SVM-RFE: Support Vector Machine with Recursive Feature Elimination; mRMR: Minimum Redundancy Maximum Relevance; TF-IDF: Term Frequency-Inverse Document Frequency; BMI: Body Mass Index; HR: Heart Rate; RR: Respiratory Rate; MAP: Mean Arterial Pressure; SBP: Systolic Blood Pressure; DBP: Diastolic Blood Pressure; SpO2: Oxygen Saturation; SCr: Serum Creatinine; BUN: Blood Urea Nitrogen; ALT: Alanine Transaminase; AST: Aspartate Aminotransferase; Alb: Albumin; HCO3: Bicarbonate; HCT: Hematocrit; HGB: Hemoglobin; Ca: Calcium; Na: Sodium; K: Potassium; RDW: Red Cell Distribution Width; Phos: Phosphate; Glu: Glucose; WBC: White Blood Cell Count; PLT: Platelets; Cl: Chloride; PT: Prothrombin Time; PTT: Partial Thromboplastin Time; INR: International Normalized Ratio; Mg: Magnesium; UA: Uric Acid; TB: Total Bilirubin; APACHE II: Acute Physiology and Chronic Health Evaluation II; SOFA: Sepsis-Related Organ Failure Assessment; APSIII: Acute Physiology Score III; SAPS II: Simplified Acute Physiology Score II; OASIS: Oxford Acute Severity of Illness Score; NGAL: Neutrophil Gelatinase-Associated Lipocalin; KIM-1: Kidney Injury Molecule-1; L-FABP: Liver-Type Fatty Acid-Binding Protein; IGFBP7: Insulin-Like Growth Factor-Binding Protein 7; IL-18: Interleukin-18; CysC: Cystatin C; MV: Mechanical Ventilation; RRT: Renal Replacement Therapy; CKD: Chronic Kidney Disease; CHF: Congestive Heart Failure; CPD: Chronic Pulmonary Disease; MI: Myocardial Infarction; CAD: Coronary Artery Disease; DM: Diabetes Mellitus; HTN: Hypertension; LD: Liver Disease; ARDS: Acute Respiratory Distress Syndrome; PaO2/FiO2: Pulse Oxygenation Index; ΔSCr: Change in Serum Creatinine; FluidBal: Net Fluid Balance; ACEI: Angiotensin-Converting Enzyme Inhibitors; ARBs: Angiotensin Receptor Blockers; NSAIDs: Nonsteroidal Anti-Inflammatory Drugs; Vanco: Vancomycin; Doc2Vec: Document to Vector Representations; ShockIdx: Shock Index; TVU: Tidal Volume Use; MetHb: Methemoglobin; COHb: Carboxyhemoglobin; MPV: Mean Platelet Volume; GHBG: Glycated Hemoglobin; CVP: Central Venous Pressure.

Table S4. TRIPOD reporting quality checklist in the selected studies.

Section/Topic	Item		Checklist Item
**Title and abstract**			
Title	1	D; V	Identify the study as developing and/or validating a multivariable prediction model, the target population, and the outcome to be predicted.
Abstract	2	D; V	Provide a summary of objectives, study design, setting, participants, sample size, predictors, outcome, statistical analysis, results, and conclusions.
**Introduction**			
Background and objectives	3a	D; V	Explain the medical context (including whether diagnostic or prognostic) and rationale for developing or validating the multivariable prediction model, including references to existing models.
	3b	D; V	Specify the objectives, including whether the study describes the development or validation of the model or both.
**Methods**			
Source of data	4a	D; V	Describe the study design or source of data (e.g., randomized trial, cohort, or registry data), separately for the development and validation data sets, if applicable.
	4b	D; V	Specify the key study dates, including start of accrual; end of accrual; and, if applicable, end of follow-up.
Participants	5a	D; V	Specify key elements of the study setting (e.g., primary care, secondary care, general population) including number and location of centres.
	5b	D; V	Describe eligibility criteria for participants.
	5c	D; V	Give details of treatments received, if relevant.
Outcome	6a	D; V	Clearly define the outcome that is predicted by the prediction model, including how and when assessed.
	6b	D; V	Report any actions to blind assessment of the outcome to be predicted.
Predictors	7a	D; V	Clearly define all predictors used in developing or validating the multivariable prediction model, including how and when they were measured.
	7b	D; V	Report any actions to blind assessment of predictors for the outcome and other predictors.
Sample size	8	D; V	Explain how the study size was arrived at.
Missing data	9	D; V	Describe how missing data were handled (e.g., complete-case analysis, single imputation, multiple imputation) with details of any imputation method.
Statistical analysis methods	10a	D	Describe how predictors were handled in the analyses.
	10b	D	Specify type of model, all model-building procedures (including any predictor selection), and method for internal validation.

	10c	V	For validation, describe how the predictions were calculated.
	10d	D; V	Specify all measures used to assess model performance and, if relevant, to compare multiple models.
	10e	V	Describe any model updating (e.g., recalibration) arising from the validation, if done.
Risk groups	11	D; V	Provide details on how risk groups were created, if done.
Development vs. validation	12	V	For validation, identify any differences from the development data in setting, eligibility criteria, outcome, and predictors.
**Results**			
Participants	13a	D; V	Describe the flow of participants through the study, including the number of participants with and without the outcome and, if applicable, a summary of the follow-up time. A diagram may be helpful.
	13b	D; V	Describe the characteristics of the participants (basic demographics, clinical features, available predictors), including the number of participants with missing data for predictors and outcome.
	13c	V	For validation, show a comparison with the development data of the distribution of important variables (demographics, predictors and outcome).
Model development	14a	D	Specify the number of participants and outcome events in each analysis.
	14b	D	If done, report the unadjusted association between each candidate predictor and outcome.
Model specification	15a	D	Present the full prediction model to allow predictions for individuals (i.e., all regression coefficients, and model intercept or baseline survival at a given time point).
	15b	D	Explain how to the use the prediction model.
Model performance	16	D; V	Report performance measures (with CIs) for the prediction model.
Model-updating	17	V	If done, report the results from any model updating (i.e., model specification, model performance).
**Discussion**			
Limitations	18	D; V	Discuss any limitations of the study (such as nonrepresentative sample, few events per predictor, missing data).
Interpretation	19a	V	For validation, discuss the results with reference to performance in the development data, and any other validation data.
	19b	D; V	Give an overall interpretation of the results, considering objectives, limitations, results from similar studies, and other relevant evidence.
Implications	20	D; V	Discuss the potential clinical use of the model and implications for future research.
**Other information**			
Supplementary information	21	D; V	Provide information about the availability of supplementary resources, such as study protocol, Web calculator, and data sets.

Funding	22	D; V	Give the source of funding and the role of the funders for the present study.

(Continued table)

Item	Research																						
	R1	R2	R3	R4	R5	R6	R7	R8	R9	R10	R11	R12	R13	R14	R15	R16	R17	R18	R19	R20	R21	R22	R23
1	√	√	√	√	√	√	√	√	√	√	√	√	√	√	√	√	√	√	√	√	√	√	√
2	×	√	√	×	√	√	×	√	√	×	√	√	×	√	×	×	×	×	×	√	√	×	×
3a	√	√	√	√	√	√	√	√	√	√	√	√	√	√	√	√	√	√	√	√	√	√	√
3b	√	√	√	√	√	√	√	√	√	√	√	√	√	√	√	√	√	√	√	√	√	√	√
4a	√	√	√	√	√	√	√	√	√	√	√	√	√	√	√	√	√	√	√	√	√	√	√
4b	×	×	×	×	√	×	×	×	×	×	×	×	×	×	×	×	×	×	×	×	×	×	×
5a	√	√	√	√	√	√	√	√	√	√	√	√	√	√	√	√	√	√	√	√	√	√	√
5b	√	√	√	√	√	√	√	√	√	√	√	√	√	√	√	√	√	√	√	√	√	√	√
5c	×	×	×	×	×	×	×	×	×	×	×	×	×	×	×	×	×	×	×	×	×	×	×
6a	√	√	√	√	√	√	√	√	√	√	√	√	√	√	√	√	√	√	√	√	√	√	√
6b	×	×	×	×	×	×	×	×	×	×	×	×	×	×	×	×	×	×	×	×	×	×	×
7a	√	√	√	√	√	√	√	√	√	√	√	√	√	√	√	√	√	√	√	√	√	√	√
7b	×	×	×	×	×	×	×	×	×	×	×	×	×	×	×	×	×	×	×	×	×	×	×
8	×	×	×	×	×	×	×	×	×	×	×	×	×	×	×	×	×	×	×	×	×	×	×
9	√	√	√	√	√	√	√	√	√	√	√	√	√	√	√	×	√	√	√	√	√	√	√
10a	×	×	√	×	√	×	√	√	√	√	×	√	×	√	√	√	√	√	√	√	√	√	×
10b	√	√	√	√	√	√	√	√	√	√	√	√	√	√	√	√	√	√	√	√	√	√	√
10c	√	√	√	√	√	√	√	√	√	√	√	√	√	√	√	√	√	√	√	√	√	√	√
10d	√	√	√	√	√	√	√	√	√	√	√	√	√	√	√	√	√	√	√	√	√	√	√
10e	√	×	√	×	×	×	√	√	√	√	√	√	×	√	×	√	√	√	×	×	√	√	×
11	×	×	×	×	×	×	×	×	×	×	×	×	×	×	×	×	×	×	×	×	×	×	×
12	√	√	√	√	√	√	√	√	√	√	√	√	√	√	√	√	√	√	√	√	√	√	√
13a	√	√	√	√	√	√	√	√	√	√	√	√	√	√	√	√	√	√	√	√	√	√	√
13b	√	√	√	√	√	√	√	√	√	√	√	√	√	√	√	√	√	√	√	√	√	√	√
13c	×	√	×	×	√	×	√	√	×	×	√	√	×	×	×	√	×	√	×	×	×	√	×

14a	√	√	√	√	√	√	√	√	√	√	√	√	√	√	√	√	√	√	√	√	√	√	√
14b	×	×	×	×	×	×	×	×	×	×	×	×	×	×	×	×	×	×	×	×	×	×	×
15a	√	√	√	√	√	√	√	√	√	√	√	√	√	√	√	√	√	√	√	√	√	√	√
15b	×	×	×	×	√	×	×	×	×	×	√	√	×	×	×	×	×	×	×	×	×	×	×
16	√	√	√	√	√	√	√	√	√	√	√	√	√	√	√	√	√	√	√	√	√	√	√
17	×	×	×	×	×	×	×	×	×	×	×	×	×	×	×	×	×	×	×	×	×	×	×
18	√	√	√	√	√	√	√	√	√	√	√	√	√	√	√	√	√	√	√	√	√	√	√
19a	×	√	×	×	√	×	√	√	×	×	√	√	×	×	×	√	×	√	×	×	×	√	×
19b	√	√	√	√	√	√	√	√	√	√	√	√	√	√	√	√	√	√	√	√	√	√	√
20	√	√	√	√	√	√	√	√	√	√	√	√	√	√	√	√	√	√	√	√	√	√	√
21	×	√	√	×	√	×	√	√	√	√	√	√	×	√	×	√	√	√	√	√	×	√	×
22	√	√	√	√	√	√	√	√	√	√	√	√	√	√	√	√	√	√	√	√	√	√	√

(Continued table)

Item	Research																						
	R24	R25	R26	R27	R28	R29	R30	R31	R32	R33	R34	R35	R36	R37	R38	R39	R40	R41	R42	R43	R44	R45	R46	R47
1	√	√	√	√	√	√	√	√	√	√	√	√	√	√	√	√	√	√	√	√	√	√	√	√
2	√	×	×	√	√	×	×	×	×	×	×	×	×	×	×	×	×	×	√	×	×	×	√	√
3a	√	√	√	√	√	√	√	√	√	√	√	√	√	√	√	√	√	√	√	√	√	√	√	√
3b	√	√	√	√	√	√	√	√	√	√	√	√	√	√	√	√	√	√	√	√	√	√	√	√
4a	√	√	√	√	√	√	√	√	√	√	√	√	√	√	√	√	√	√	√	√	√	√	√	√
4b	×	×	×	×	×	×	×	×	×	×	×	×	×	×	×	×	×	×	×	×	×	×	×	√
5a	√	√	√	√	√	√	√	√	√	√	√	√	√	√	√	√	√	√	√	√	√	√	√	√
5b	√	√	√	√	√	√	√	√	√	√	√	√	√	√	√	√	√	√	√	√	√	√	√	√
5c	×	×	×	×	×	×	×	×	×	×	×	×	×	×	×	×	×	×	×	×	×	×	×	×
6a	√	√	√	√	√	√	√	√	√	√	√	√	√	√	√	√	√	√	√	√	√	√	√	√
6b	×	×	×	×	×	×	×	×	×	×	×	×	×	×	×	×	×	×	×	×	×	×	×	×
7a	√	√	√	√	√	√	√	√	√	√	√	√	√	√	√	√	√	√	√	√	√	√	√	√
7b	×	×	×	×	×	×	×	×	×	×	×	×	×	×	×	×	×	×	×	×	×	×	×	×
8	×	×	×	×	×	×	×	×	×	×	×	×	×	×	×	×	×	×	×	×	×	×	×	×
9	√	√	√	√	√	×	√	√	√	√	√	√	√	√	√	√	√	×	√	√	×	√	×	×

10a	×	√	×	×	√	×	√	√	√	×	√	×	√	×	×	√	√	√	√	√	√	√	√	×
10b	√	√	√	√	√	√	√	√	√	√	√	√	√	√	√	√	√	√	√	√	√	√	√	√
10c	√	√	√	√	√	√	√	√	√	√	√	√	√	√	√	√	√	√	√	√	√	√	√	√
10d	√	√	√	√	√	√	√	√	√	√	√	√	√	√	√	√	√	√	√	√	√	√	√	√
10e	√	×	×	√	√	√	×	×	√	×	√	×	√	×	√	×	√	√	×	×	×	√	√	×
11	×	×	×	×	×	×	×	×	×	×	×	×	×	×	×	×	×	×	×	×	×	×	×	×
12	√	√	√	√	√	√	√	√	√	√	√	√	√	√	√	√	√	√	√	√	√	√	√	√
13a	√	√	√	√	√	√	√	√	√	√	√	√	√	√	√	√	√	√	√	√	√	√	√	√
13b	√	√	√	√	√	√	√	√	√	√	√	√	√	√	√	√	√	√	√	√	√	√	√	√
13c	×	√	√	√	×	×	×	×	√	×	×	×	×	×	×	√	×	×	×	×	×	×	×	×
14a	√	√	√	√	√	√	√	√	√	√	√	√	√	√	√	√	√	√	√	√	√	√	√	√
14b	×	×	×	×	×	×	×	×	×	×	×	×	×	×	×	×	×	×	×	×	×	×	×	×
15a	√	√	√	√	√	√	√	√	√	√	√	√	√	√	√	√	√	√	√	√	√	√	√	√
15b	×	√	×	×	×	×	×	×	×	×	×	×	×	×	×	×	×	√	×	×	×	×	×	×
16	√	√	√	√	√	√	√	√	√	√	√	√	√	√	√	√	√	√	√	√	√	√	√	√
17	×	×	×	×	×	×	×	×	×	×	×	×	×	×	×	×	×	×	×	×	×	×	×	×
18	√	√	√	√	√	√	√	√	√	√	√	√	√	√	√	√	√	√	√	√	√	√	√	√
19a	×	√	√	√	×	×	×	×	√	×	×	×	×	×	×	√	×	√	×	×	×	×	×	×
19b	√	√	√	√	√	√	√	√	√	√	√	√	√	√	√	√	√	√	√	√	√	√	√	√
20	√	√	√	√	√	√	√	√	√	√	√	√	√	√	√	√	√	√	√	√	√	√	√	√
21	√	√	×	√	√	×	√	×	√	×	√	√	√	×	×	√	√	√	√	×	×	×	√	×
22	√	√	√	√	√	√	√	√	√	√	√	√	√	√	√	√	√	√	√	√	√	√	√	√

Items relevant only to the development of a prediction model are denoted by D, items relating solely to a validation of a prediction model are denoted by V, and items relating to both are denoted D; V. We recommend using the TRIPOD Checklist in conjunction with the TRIPOD Explanation and Elaboration document. "√" means "Yes", "×" means "No". The R (R1-R47) in the table indicates the research included in this review, and its order corresponds to the citation order of reference in the Supplementary Materials file and Table S2-S3.

Reference

1. Zhang X, Liang X, Fu Z, Zhou Y, Fang Y, Liu X, Yuan Q, Liu R, Hong Q, Liu C: **Interpretable machine learning model for early prediction of acute kidney injury in patients with rhabdomyolysis**. *Emerg Crit Care Med* 2024.
2. Yang M, Liu S, Hao T, Ma C, Chen H, Li Y, Wu C, Xie J, Qiu H, Li J *et al*: **Development and validation of a deep interpretable network for continuous acute kidney injury prediction in critically ill patients**. *Artificial Intelligence in Medicine* 2024, **149**.
3. Tu H, Su J, Gong K, Li Z, Yu X, Xu X, Shi Y, Sheng J: **A dynamic model to predict early occurrence of acute kidney injury in ICU hospitalized cirrhotic patients: a MIMIC database analysis**. *BMC Gastroenterology* 2024, **24**(1).
4. Tan Y, Dede M, Mohanty V, Dou J, Hill H, Bernstam E, Chen K: **Forecasting acute kidney injury and resource utilization in ICU patients using longitudinal, multimodal models**. *Journal of Biomedical Informatics* 2024, **154**.
5. Sun T, Yue X, Zhang G, Lin Q, Chen X, Huang T, Li X, Liu W, Tao Z: **AKIMLpred: An interpretable machine learning model for predicting acute kidney injury within seven days in critically ill patients based on a prospective cohort study**. *Clinica Chimica Acta* 2024, **559**.
6. Shi J, Han H, Chen S, Liu W, Li Y: **Machine learning for prediction of acute kidney injury in patients diagnosed with sepsis in critical care**. *PLoS ONE* 2024, **19**(4 April).
7. Lyu X, Fan B, Hüser M, Hartout P, Gumbsch T, Faltys M, Merz TM, Rätsch G, Borgwardt K: **An empirical study on KDIGO-defined acute kidney injury prediction in the intensive care unit**. *Bioinformatics* 2024, **40**:i247-i256.
8. Lu X, Chen Y, Zhang G, Zeng X, Lai L, Qu C: **Application of interpretable machine learning algorithms to predict acute kidney injury in patients with cerebral infarction in ICU**. *J Stroke Cerebrovasc Dis* 2024, **33**(7).
9. Liu X, Fang M, Wang K, Zhu J, Chen Z, He L, Liang S, Deng Y, Chen C: **Machine learning-based model to predict severe acute kidney injury after total aortic arch replacement for acute type A aortic dissection**. *Heliyon* 2024, **10**(13).
10. Lin S, Lu W, Wang T, Wang Y, Leng X, Chi L, Jin P, Bian J: **Predictive model of acute kidney injury in critically ill patients with acute pancreatitis: a machine learning approach using the MIMIC-IV database**. *Renal Failure* 2024, **46**(1).
11. Li M, Han S, Liang F, Hu C, Zhang B, Hou Q, Zhao S: **Machine Learning for Predicting Risk and Prognosis of Acute Kidney Disease in Critically Ill Elderly Patients During Hospitalization: Internet-Based and Interpretable Model Study**. *Journal of Medical Internet Research* 2024, **26**.
12. Zheng L, Lin Y, Fang K, Wu J, Zheng M: **Derivation and validation of a risk score to predict acute kidney injury in critically ill cirrhotic patients**. *Hepatology Research* 2023, **53**(8):701-712.
13. Zhang Y, Zhang Z, Liu X, Zha L, Fengcong, Su X, Zhao B, Hu L, Hu P: **A Deep Learning Approach Incorporating Data Missing Mechanism in Predicting Acute Kidney Injury in ICU**. In: *19th International Conference on Advanced Intelligent Computing Technology and Applications (ICIC): 2023 Aug 10-13 2023; Zhengzhou, PEOPLES R CHINA*; 2023: 335-346.

14. Wu M, Jiang X, Du K, Xu Y, Zhang W: **Ensemble machine learning algorithm for predicting acute kidney injury in patients admitted to the neurointensive care unit following brain surgery**. *Scientific Reports* 2023, **13**(1).
15. Wu K, Chen EH, Wirth F, Vitanova K, Lange R, Burschka D: **Continuous Risk Estimation of Acute Kidney Failure with Dense Temporal Data for ICU Patients**. In: *2023 45th Annual International Conference of the IEEE Engineering in Medicine & Biology Society (EMBC): 24-27 July 2023 2023*; 2023: 1-5.
16. Shi H, Shen Y, Li L: **Early prediction of acute kidney injury in patients with gastrointestinal bleeding admitted to the intensive care unit based on extreme gradient boosting**. *Frontiers in Medicine* 2023, **10**.
17. Persson I, Grünwald A, Morvan L, Becedas D, Arlbrandt M: **A Machine Learning Algorithm Predicting Acute Kidney Injury in Intensive Care Unit Patients (NAVOY Acute Kidney Injury): Proof-of-Concept Study**. *JMIR Formative Research* 2023, **7**(1).
18. Peng C, Yang F, Li L, Peng L, Yu J, Wang P, Jin Z: **A Machine Learning Approach for the Prediction of Severe Acute Kidney Injury Following Traumatic Brain Injury**. *Neurocritical Care* 2023, **38**(2):335-344.
19. Pan P, Liu Y, Xie F, Duan Z, Li L, Gu H, Xie L, Lu X, Su L: **Significance of platelets in the early warning of new-onset AKI in the ICU by using supervise learning: a retrospective analysis**. *Renal Failure* 2023, **45**(1).
20. Luo Y, Ye W, Sun Y, Bao H, Liu H: **Development and Comparative Analysis of an Early Prediction Model for Acute Kidney Injury within 72-Hours Post-ICU Admission Using Evidence from the MIMIC-III Database**. *Discovery Medicine* 2023, **35**(177):623-631.
21. Jiang Z, An X, Li Y, Xu C, Meng H, Qu Y: **Construction and validation of a risk assessment model for acute kidney injury in patients with acute pancreatitis in the intensive care unit**. *BMC Nephrology* 2023, **24**(1).
22. Huang S, Teng Y, Du J, Zhou X, Duan F, Feng C: **Internal and external validation of machine learning–assisted prediction models for mechanical ventilation–associated severe acute kidney injury**. *Australian Critical Care* 2023, **36**(4):604-612.
23. Begum MF, Narayan S: **A pattern mixture model with long short-term memory network for acute kidney injury prediction**. *Journal of King Saud University-Computer and Information Sciences* 2023, **35**(4):172-182.
24. Yue S, Li S, Huang X, Liu J, Hou X, Zhao Y, Niu D, Wang Y, Tan W, Wu J: **Machine learning for the prediction of acute kidney injury in patients with sepsis**. *J Transl Med* 2022, **20**(1):215.
25. Zhang L, Wang Z, Zhou Z, Li S, Huang T, Yin H, Lyu J: **Developing an ensemble machine learning model for early prediction of sepsis-associated acute kidney injury**. *iScience* 2022, **25**(9):104932.
26. Zeng G, Zhuang J, Huang H, Gao Y, Liu Y, Yu X: **Continuous Prediction of Acute Kidney Injury from Patients with Sepsis in ICU Settings: A Sequential Transduction Model Based on Attention**. In: *ACM International Conference Proceeding Series: 2022*: Association for Computing Machinery; 2022: 31-37.
27. Zhang X, Chen S, Lai K, Chen Z, Wan J, Xu Y: **Machine learning for the prediction of acute kidney injury in critical care patients with acute cerebrovascular disease**. *Ren Fail* 2022, **44**(1):43-53.
28. Wen K, Huang Y, Guo Q, Wu T, Liu J, Zheng Y, Zhou S, Geng D: **Predicting Risk Factors of Acute Kidney Injury in the First 7 Days after Admission: Analysis of a Group of Critically Ill Patients**. *Cardiovascular Therapeutics* 2022, **2022**.

29. Vagliano I, Hsu WH, Schut MC: **Machine Learning, Clinical Notes and Knowledge Graphs for Early Prediction of Acute Kidney Injury in the Intensive Care**. In: *Studies in Health Technology and Informatics: 2022*: IOS Press BV; 2022: 329-332.
30. Gao W, Wang J, Zhou L, Luo Q, Lao Y, Lyu H, Guo S: **Prediction of acute kidney injury in ICU with gradient boosting decision tree algorithms**. *Computers in Biology and Medicine* 2022, **140**.
31. Fathima Begum M, Narayan S: **A Machine Learning Model for Acute Kidney Injury Prediction with Novel Kidney Biomarkers**. In: *Proceedings - 2nd International Conference on Next Generation Intelligent Systems, ICNGIS 2022: 2022*: Institute of Electrical and Electronics Engineers Inc.; 2022.
32. Cai D, Xiao T, Zou A, Mao L, Chi B, Wang Y, Wang Q, Ji Y, Sun L: **Predicting acute kidney injury risk in acute myocardial infarction patients: An artificial intelligence model using medical information mart for intensive care databases**. *Frontiers in Cardiovascular Medicine* 2022, **9**.
33. Qian Q, Wu J, Wang J, Sun H, Yang L: **Prediction models for aki in icu: A comparative study**. *International Journal of General Medicine* 2021, **14**:623-632.
34. Luo X-Q, Yan P, Zhang N-Y, Luo B, Wang M, Deng Y-H, Wu T, Wu X, Liu Q, Wang H-S *et al*: **Machine learning for early discrimination between transient and persistent acute kidney injury in critically ill patients with sepsis**. *Scientific Reports* 2021, **11**(1).
35. Le S, Allen A, Calvert J, Palevsky PM, Braden G, Patel S, Pellegrini E, Green-Saxena A, Hoffman J, Das R: **Convolutional Neural Network Model for Intensive Care Unit Acute Kidney Injury Prediction**. *Kidney International Reports* 2021, **6**(5):1289-1298.
36. Gong K, Lee HK, Yu K, Xie X, Li J: **A prediction and interpretation framework of acute kidney injury in critical care**. *Journal of Biomedical Informatics* 2021, **113**.
37. Chiorean IA, Amico B, Combi C, Holmes JH: **A Reproducible ETL Approach for Window-based Prediction of Acute Kidney Injury in Critical Care Unit and Some Preliminary Results with Support Vector Machines**. In: *2021 IEEE International Conference on Bioinformatics and Biomedicine (BIBM): 9-12 Dec. 2021 2021*; 2021: 3532-3539.
38. Alfieri F, Ancona A, Tripepi G, Crosetto D, Randazzo V, Paviglianiti A, Pasero E, Vecchi L, Cauda V, Fagugli RM: **A deep-learning model to continuously predict severe acute kidney injury based on urine output changes in critically ill patients**. *Journal of Nephrology* 2021, **34**(6):1875-1886.
39. Wang Y, Wei Y, Yang H, Li J, Zhou Y, Wu Q: **Utilizing imbalanced electronic health records to predict acute kidney injury by ensemble learning and time series model**. *BMC Medical Informatics and Decision Making* 2020, **20**(1).
40. Rank N, Pfahringer B, Kempfert J, Stamm C, Kühne T, Schoenrath F, Falk V, Eickhoff C, Meyer A: **Deep-learning-based real-time prediction of acute kidney injury outperforms human predictive performance**. *npj Digital Medicine* 2020, **3**(1).
41. Matsuura R, Iwagami M, Moriya H, Ohtake T, Hamasaki Y, Nangaku M, Doi K, Kobayashi S, Noiri E: **A Simple Scoring Method for Predicting the Low Risk of Persistent Acute Kidney Injury in Critically Ill Adult Patients**. *Scientific Reports* 2020, **10**(1).
42. Zimmerman LP, Reyfman PA, Smith ADR, Zeng Z, Kho A, Sanchez-Pinto LN, Luo Y: **Early prediction of acute kidney injury following ICU admission using a multivariate panel of physiological measurements**. *BMC Med Inform Decis Mak* 2019, **19**(Suppl 1):16.
43. Wang Y, Wei Y, Wu Q, Yang H, Li J: **An Acute Kidney Injury Prediction Model Based on Ensemble Learning Algorithm**. In: *2019 10th International Conference on Information Technology in Medicine and Education (ITME): 23-25 Aug. 2019 2019*; 2019: 18-22.

44. Parreco J, Soe-Lin H, Parks JJ, Byerly S, Chatoor M, Buicko JL, Namias N, Rattan R: **Comparing machine learning algorithms for predicting acute kidney injury**. *American Surgeon* 2019, **85**(7):725-729.
45. Li Y, Yao L, Mao C, Srivastava A, Jiang X, Luo Y: **Early Prediction of Acute Kidney Injury in Critical Care Setting Using Clinical Notes**. In: *Proceedings - 2018 IEEE International Conference on Bioinformatics and Biomedicine, BIBM 2018: 2019*: Institute of Electrical and Electronics Engineers Inc.; 2019: 683-686.
46. Chiofolo C, Chbat N, Ghosh E, Eshelman L, Kashani K: **Automated Continuous Acute Kidney Injury Prediction and Surveillance: A Random Forest Model**. *Mayo Clinic Proceedings* 2019, **94**(5):783-792.
47. Mao Y, Qin Z-H: **Risk factors of acute kidney injury and dialysis among patients attending intensive care units in China**. *International Journal of Clinical and Experimental Medicine* 2017, **10**(8):12056-12067.
